# *SPNS1* variants cause multiorgan disease and implicate lysophospholipid transport as critical for mTOR-regulated lipid homeostasis

**DOI:** 10.1172/JCI193099

**Published:** 2025-07-03

**Authors:** Menglan He, Mei Ding, Michaela Chocholouskova, Cheen Fei Chin, Martin Engvall, Helena Malmgren, Matias Wagner, Marlen C. Lauffer, Jacob Heisinger, May Christine V. Malicdan, Valerie Allamand, Madeleine Durbeej, Angelica Delgado Vega, Thomas Sejersen, Ann Nordgren, Federico Torta, David L. Silver

**Affiliations:** 1 Signature Research Program in Cardiovascular and Metabolic Disorders, Duke-NUS Medical School, Singapore.; 2 Singapore Lipidomics Incubator (SLING), Life Sciences Institute, and; 3 Precision Medicine Translational Research Programme and Department of Biochemistry, YLL School of Medicine, National University of Singapore, Singapore.; 4 Centre for Inherited Metabolic Diseases, Karolinska University Hospital, Stockholm, Sweden.; 5 Department of Molecular Medicine and Surgery, Karolinska Institutet, Stockholm, Sweden.; 6 Department of Clinical Genetics and Genomics, Karolinska University Hospital, Solna, Stockholm, Sweden.; 7 Institute of Human Genetics, Klinikum Rechts der Isar, School of Medicine, Technical University of Munich, Munich, Germany; Institute for Neurogenomics, Helmholtz Zentrum München, Neuherberg, Germany; Division of Paediatric Neurology, Developmental Neurology, and Social Pediatrics, Dr von Hauner Children’s Hospital, Munich, Germany.; 8 Dutch Center for RNA Therapeutics, Department of Human Genetics, Leiden University Medical Center, Leiden, The Netherlands.; 9 Hospital of the Brothers of Mercy Eisenstadt, Eisenstadt, Austria.; 10 National Institutes of Health (NIH) Undiagnosed Disease Program, National Human Genome Research Institute, Human Biochemical Genetics Section, Medical Genetics Branch, National Human Genome Research Institute, NIH, Bethesda, Maryland, USA.; 11 Sorbonne Université, INSERM, Institut de Myologie, Center de Recherche en Myologie, Paris, France.; 12 Department of Experimental Medical Science, Lund University, Lund, Sweden.; 13 Department of Women′s and Children′s Health, Karolinska Institutet, Stockholm, Sweden.; 14 Department of Child Neurology, Karolinska University Hospital, Astrid Lindgren Children’s Hospital, Stockholm, Sweden.; 15 Center for Neuromusculoskeletal Restorative Medicine, Hong Kong Science Park, Shatin, New Territories, Hong Kong.; 16 Department of Laboratory Medicine, Institute of Biomedicine, University of Gothenburg, Gothenburg, Sweden.; 17 Department of Clinical Genetics and Genomics, Sahlgrenska University Hospital, Gothenburg, Sweden.

**Keywords:** Cell biology, Metabolism, Cholesterol, Lipidomics, Lysosomes

## Abstract

SPNS1 is a lysosomal transporter that mediates the salvage of lysoglycerophospholipids, the degradative products of lysosomal phospholipid catabolism. However, an understanding of the role of lysolipid transport and salvage in regulating cellular lipid homeostasis and in disease is lacking. Here, we identified members of 2 families with biallelic *SPNS1* loss-of-function variants, who presented primarily with progressive liver and striated muscle injury. Patients’ fibroblasts accumulated lysophospholipids including lysoplasmalogens and cholesterol in lysosomes with reduced cellular plasmalogens. Notably, SPNS1 deficiency resulted in reduced biogenesis of cytosolic lipid droplets containing triglycerides and cholesteryl esters. Mechanistically, we found that lysophospholipids transported by SPNS1 into the cytosol quantitatively contributed to triglyceride synthesis, whereas lysosomal buildup of lyso-ether-phospholipid inhibited lysosomal cholesterol egress, effects that were enhanced with inhibition of mTOR. These findings support a gene-disease association and reveal connectivity between lysosomal transport of lysophospholipids and storage of reserve cellular energy as triglycerides and the regulation of cholesterol homeostasis, processes that become important under nutrient limitation.

## Abstract 2

Lysosomal lysophospholipid transport is critical for the regulation of cellular lipid homeostasis

## Graphical abstract

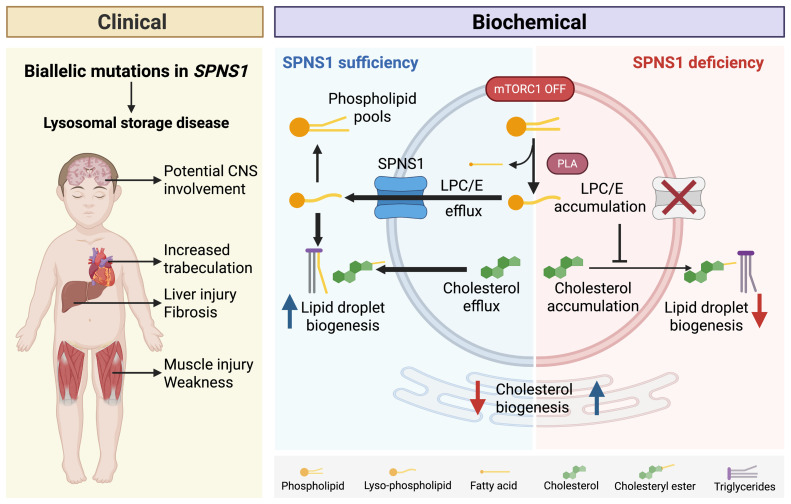

## Introduction

Lysosomes are degradative organelles that receive macromolecules from endocytosis, autophagy, and phagocytosis. In addition to protein degradation leading to amino acid recycling, lysosomes also break down lipids and transport these back into biosynthetic pathways in the cytoplasm. One of the most well-understood lysosomal lipid transport pathways is the transport of cholesterol by the Niemann-Pick C1 (NPC1) and NPC2 proteins (1, 2). Biallelic loss of either NPC1 or NPC2 leads to Niemann-Pick disease type C (NPC), a lysosomal storage disease affecting multiple organs such as the brain, liver, and spleen that is associated with lysosomal accumulation of cholesterol and sphingolipids (3–5). Transport of cholesterol from lysosomes distributes it to the plasma membrane and acts to negatively regulate de novo cholesterol biosynthesis in the endoplasmic reticulum (ER) by suppressing the processing of SREBP2, the master regulator of cholesterogenic gene expression (6, 7). Thus, cholesterol egress from lysosomes is critical for regulating cellular cholesterol homeostasis, and a defect in this process is thought to be the major driver of NPC disease. In addition to lysosomal cholesterol egress, the glycerophospholipids phosphatidylcholine (PC) and phosphatidylethanolamine (PE) are the main lipid components of cellular membranes that continuously traffic through lysosomes. Within the lysosomal lumen, PC and PE are degraded by phospholipases such as PLA2G15 to release lyso-phosphatidylcholine (LPC), lyso-phosphatidylethanolamine (LPE), and fatty acids (8, 9). Because of the zwitterionic nature of LPC and LPE lipids, we predicted that their egress from lysosomes would require a transporter (10). Indeed, we and other groups identified SPNS1, a ubiquitously expressed lysosomal transmembrane protein belonging to the major facilitator superfamily, as a proton-dependent transporter of LPC and LPE (10–12). We demonstrated that LPC transported out of lysosomes was re-acylated in the ER, constituting a lysosomal salvage pathway for PC. SPNS1-mediated phospholipid salvage becomes essential for cell growth and survival under conditions of choline deficiency or blockade in cellular choline uptake (12, 13), providing further evidence for the importance of the SPNS1/LPC salvage pathway in cellular physiology. Spns1 deficiency in mice is embryonically lethal, whereas knockdown or genetic deletion of *Spns1* in adult mice results in pathologies including liver inflammation, demyelination in brain, and skeletal muscle atrophy (10, 11, 14, 15). Such pathological changes are associated with enlarged lysosomes and impaired autophagy, similar to the zebrafish models of SPNS1 deficiency (10, 11, 16, 17). The role of SPNS1 in human health and disease is less clear. A recent report identified 3 patients from 1 family with a homozygous variant of *SPNS1* (c.C884T:p.P295L) who presented with neurodevelopmental delay (11), but without the reported liver or muscle pathologies. A direct link between this variant and cellular accumulation of LPC and LPE was not reported. Therefore, our understanding of the importance of *SPNS1* in human physiology is limited by a lack of definitively identified pathogenic variants of *SPNS*1 that lead to human disease.

In addition to LPC and LPE, sphingolipid metabolites including hexosylceramides and sphingosine also accumulate in lysosomes of mice in models of Spns1 deficiency (10, 11). Notably, with liver-specific knockdown of *Spns1*, we found an increase in lysosomal levels of cholesterol-biochemical phenotypes reminiscent of NPC disease. The accumulation of lipids not transported by SPNS1 suggested that lysosomal accumulation of LPC and LPE could potentially affect the homeostasis of other lipids in the cells. mTOR is a key nutrient sensor that responds to growth hormone, insulin, and lysosomal amino acid flux to regulate anabolic processes such as protein translation and lipogenesis (18–20). Recently, inhibition of mTOR has been shown to additionally regulate lysosomal phospholipid catabolism to produce LPC and fatty acids, the latter of which was demonstrated to contribute to the synthesis of cytosolic triglyceride lipid droplets (LDs) as an adaptation to nutrient deficiency (21). However, the contribution of lysosomal LPC to cellular lipid homeostasis in conditions of mTOR inhibition remains unknown.

In this study, we expand upon the clinical and mutational spectrum of *SPNS1*-associated disease and identify the underlying mechanisms by which SPNS1 deficiency can negatively affect lysosomal and cellular lipid homeostasis.

## Results

### Biallelic SPNS1 loss-of-function variants lead to a multiorgan disease

#### Family A.

The proband (A.II.1, male, 16 years old, Supplemental Figure 1A; supplemental material available online with this article; https://doi.org/10.1172/JCI193099DS1) and his younger brother (A.II.2, male, 8 years old) both presented with prolonged, transient neonatal unconjugated hyperbilirubinemia followed by persistently elevated transaminases, serum creatine kinase, and myoglobin levels since 6 months and 12 months of age, respectively. There was no family history of liver, muscle, or other metabolic disorders. Echocardiography showed increased trabeculation of the left ventricle in both brothers and discrete left ventricular hypertrophy in the proband. Motor milestones were achieved in the lower part of the normal spectrum in both brothers. At 4 years of age, gross motor function was slightly impaired in the proband, with calf pain and reduced ankle range of motion. At his last gross motor assessment at the age of 15 years, mild proximal/axial weakness was noted, particularly in the abdomen and upper extremities. He scored 93 of 96 on the motor function measure but was unable to perform push-ups and could only do a few sit-ups with great difficulty. Both brothers are ambulatory but easily fatigued. Speech development was slightly delayed in both brothers. The proband was diagnosed with attention deficit disorder at the age of 13 years. Neuropsychological assessments of cognition using the Wechsler Intelligence Scale for Children at 8 and 13 years of age showed average verbal abilities but reduced nonverbal abilities. Key clinical and laboratory findings are summarized in Supplemental Table 1.

Whole-genome sequencing and analysis of the 2 affected brothers and their parents showed that the siblings were both compound heterozygous for 2 variants in the *SPNS1* gene (NM_032038); c.(1247C>G), p[Ser416Cys] (referred to as S416C), and c.(143_146dup), p[Ile50Alafs*48] (referred to as 143_146 dup). The father was the heterozygous carrier of the c.1247C>G variant, and the mother was the heterozygous carrier of the c.143_146dup variant in the *SPNS1* gene. Two healthy sisters did not harbor any of the variants in the *SPNS1* gene. In addition, a hemizygous variant in the *PRDX4* gene was detected in both patients, and the mother was a heterozygous carrier for this variant. However, this candidate gene was excluded, as the healthy grandfather of the patients was hemizygous for this variant.

#### Family B.

We screened our in-house database (22) comprising over 20,000 exome datasets for individuals with biallelic variants in *SPNS1* and identified patient 3, who is homozygous for the variant NM_032038.3:c.860C>T, p.(Thr287Met). Patient 3, an 8-year-old male, was born to consanguineous parents and presented with elevated transaminase and failure to thrive at 2.5 years of age. Liver transaminase, lactate dehydrogenase, and serum creatine kinase levels had persistently remained elevated previously. He was diagnosed with global development delay at 3 years of age. The patient had neonatal cardiac abnormalities including cardiomegaly, large coronary arteries, and a small aortic isthmus, but a subsequent cardiac ultrasound had inconspicuous findings. Key clinical and laboratory findings are summarized in Supplemental Table 1.

### SPNS1 variants result in loss of function of LPC transport

**Figure 1:**
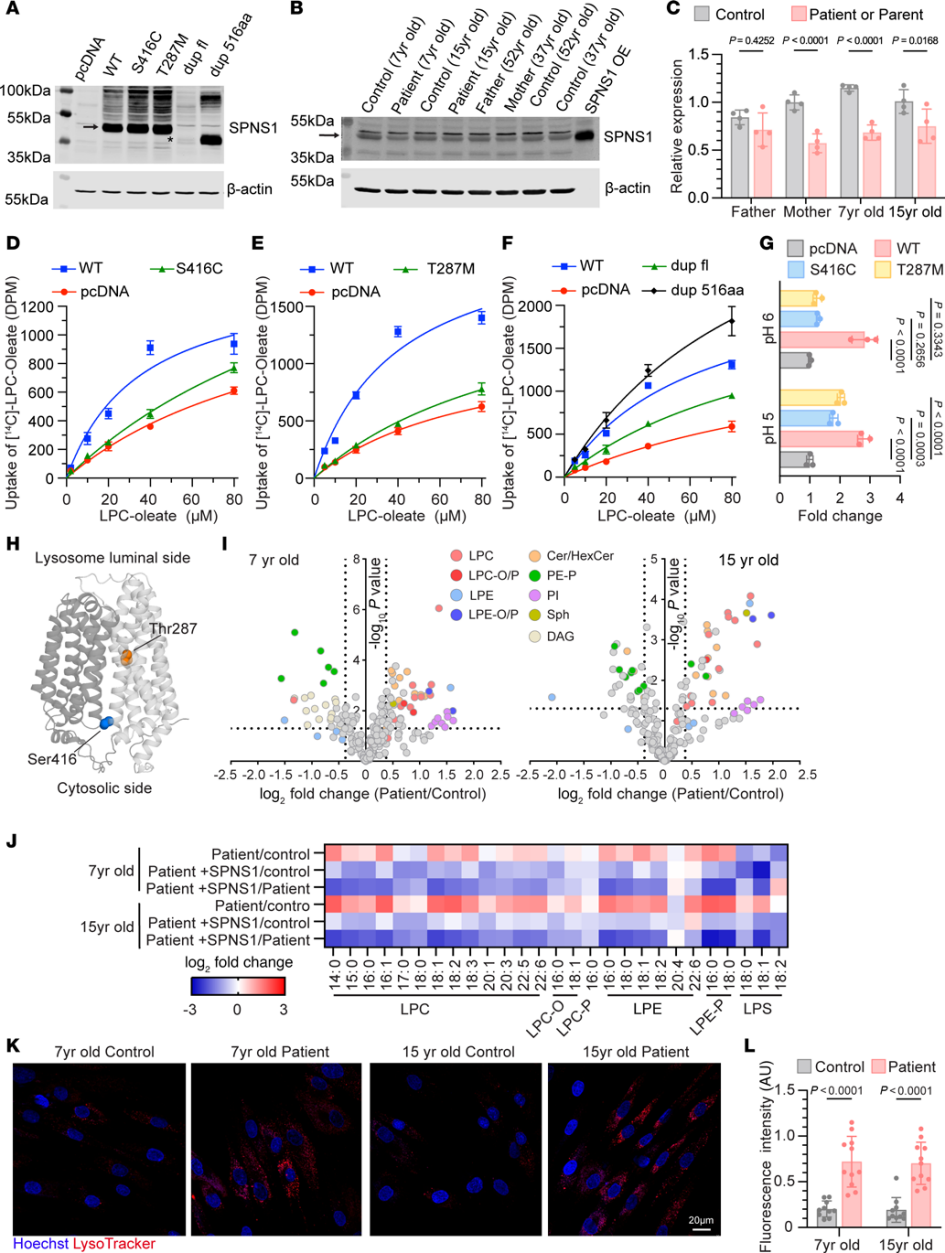
Characterization of SPNS1 mutations identified in patients. (**A**) Immunoblotting of ectopically expressed WT SPNS1 (WT), p.Ser416Cys (S416C), p.Thr287Met (T287M), c.143_146dupAGCG (dup fl), and the duplication mutant starting from the alternative ATG (dup 516 aa) in HEK293 cell. The arrow indicates SPNS1, and the asterisk indicates the dup 516 aa SPNS1 that migrates at a lower molecular weight. (**B**) Immunoblotting of endogenous SPNS1 from patients’ and parents’ fibroblasts with their respective age-matched controls. Cell lysate from HEK293 cells overexpressing WT SPNS1 (SPNS1 OE) was used as a control. The arrow indicates SPNS1. (**C**) Quantification of SPNS1 protein levels normalized to β-actin in **B**. (**D**–**F**) Concentration-dependent transport of [^14^C]-LPC-oleate by HEK293 cells overexpressing S416C (**D**), T287M (**E**), dup fl, and dup 516 aa (**F**) as compared with overexpressing WT and vector control (pcDNA) cells over 30 minutes at pH 6 extracellular buffer. (**G**) [^14^C]-LPC-oleate transport activity of WT and mutant SPNS1 at pH 6 and pH 5. (**H**) Alpha-Fold model of SPNS1 with Ser416 and Thr287 residues highlighted. (**I**) Volcano plots showing lipidomic changes in patients’ fibroblasts relative to their age-matched controls. Lipids are color-coded according to lipid species. (**J**) Heatmap representation of log_2_-transformed fold change in lipid concentration for patients’ and age-matched control fibroblasts transduced with lentivirus carrying either WT *SPNS1* construct or vector control. (**K**) LysoTracker staining of patients’ and control fibroblasts. Red: LysoTracker; blue: Hoechst. Scale bar: 20 μm. (**L**) Quantification of LysoTracker fluorescence intensity of individual cells shown in **K**. *n* = at least 10 cells from 5 different images. Each data point represents 1 cell. *n* = 4 replicates (**C**) and *n* = 3 replicates (**D**–**G**, **I**, and **J**). Data are presented as the mean ± SD. Statistical significance was determined by 2-sided, unpaired Student’s *t* test (**I** and **J**), 2-way ANOVA with Šídák’s test (**C**) and (**L**), and 2-way ANOVA with Dunnett’s test (**G**). *P* values for **I** and **J** are presented in the Supplemental Data.

To understand the functional effect of the *SPNS1* variants identified in the patients, we introduced the mutations encoding S416C, T287M, and 143_146dup into the human cDNA of *SPNS1* and induced overexpression of these in HEK293 cells. We found that S416C and T287M were expressed at levels similar to those for WT SPNS1, whereas the full-length 143_146dup (dup fl) variant resulted in a poorly expressed protein migrating at a lower molecular weight (Figure 1A). This residual expression was possibly due to usage of an alternative start codon located 38 bp downstream of the original start codon that is predicted to result in a smaller protein (referred to as dup 516 aa) with a new N-terminal sequence (Supplemental Figure 1, B and C), consistent with the smaller molecular weight detected by Western blot analysis (Figure 1A). In fact, when we induced overexpression of the c.143_146dup variant that only had the alternative start codon, the modified protein dup 516 aa was expressed at a level similar to that of WT SPNS1 (Figure 1A). In fibroblasts from patients A.II.1 and A.II.2, the protein levels of SPNS1 were reduced compared with expression levels in their age-matched controls (Figure 1, B and C). Such a reduction was likely caused by low expression of the 143_146dup variant because the mother, who is the carrier of this variant, also had a similar reduction in SPNS1 protein levels, while SPNS1 expression levels in the father’s fibroblasts were comparable to those of the age-matched controls (Figure 1, B and C). To test the functionality of *SPNS1* variants, we used a cell-surface LPC transport assay we previously developed, taking advantage of a cellular phenomenon that lysosomal membrane proteins traffic through the plasma membrane before entering the endocytic pathway (23). This assay works by overexpression of WT and variant constructs at the cell surface and quantifying the uptake of [^14^C]-LPC-oleate at an extracellular acidic pH to mimic the lysosomal environment. We first confirmed that S416C and T287M mutants were present at levels comparable to those of WT SPNS1 at the plasma membrane by immunofluorescence and cell-surface biotinylation upon overexpression (Supplemental Figure 1, D and E). The plasma membrane levels of the 143_146dup (dup fl) mutant were low, consistent with its poor cellular expression levels (Supplemental Figure 1, D and E). We found that, while cells overexpressing WT SPNS1 showed a dose-dependent uptake of [^14^C]-LPC-oleate, the S416C and T287M variants had reduced transport activity (Figure 1, D and E), suggestive of a partial loss of function. In contrast, the dup fl variant showed reduced transport activity, while the dup 516 aa construct showed higher transport activity than the WT (Figure 1F), indicating that the low transport activity of the dup fl variant was due to its poor expression. We also noticed that the transport activity of S416C and T287M variants was enhanced when the assay was performed at a lower pH (Figure 1G). This suggests that in the lysosome, where the luminal pH is close to 4.5, these mutants are partially functional. This is consistent with SPNS1 activity being pH dependent (10). To test the functionality of these 2 variants within the lysosome, we integrated cDNAs encoding WT, S416C, and T287M into the *AAVS1* locus of HEK293T *SPNS1*-KO cells. We confirmed that both variants were expressed at a level similar to that of WT SPNS1 (Supplemental Figure 1F) and localized to lysosomes (Supplemental Figure 1G). The 2 patients’ variants were able to reduce cellular LPC accumulation to a similar extent as that observed with WT SPNS1 (Supplemental Figure 1H). We also observed that the expression of S416C and T287M in the rescue cell lines was higher than endogenous SPNS1 expression in HEK293T cells (Supplemental Figure 1F), further supporting the conclusion that these *SPNS1* variants are partially active and their elevated expression in lysosomes can reduce LPC accumulation in *SPNS1*-KO cells. An examination of an AlphaFold-predicted SPNS1 structure showed that Ser416 is located near the cytosolic end of the transport cleft, whereas Thr287 is in the transport cleft (Figure 1H). Both residues are highly conserved across the species (Supplemental Figure 1I) and expected to play a role in transport function through either direct substrate interactions or by participating in conformational changes between the N- and C-terminal halves of SPNS1.

We similarly conducted biochemical analysis of the previously reported P295L variant of SPNS1 (11) and found that P295L was expressed at a lower level than WT SPNS1 upon transient overexpression (Supplemental Figure 1J) and had minimal transport activity (Supplemental Figure 1, K and L). Notably, transiently overexpressed P295L showed mainly a reticular staining pattern and consequently had lower plasma membrane levels compared with WT SPNS1 as determined by cell-surface biotinylation (Supplemental Figure 1, D and E), likely explaining its lack of transport activity in this assay and in the previous report (11). To determine the function of the P295L variant at the lysosome level, we similarly integrated the P295L variant into the *AAVS1* locus. In the rescued line, P295L localized to lysosomes and was expressed at a lower level than was WT SPNS1 but at a level similar to that of endogenous SPNS1 (Supplemental Figure 1, F and G). Expression of P295L rescued LPC accumulation in *SPNS1*-KO cells despite the lower expression of P295L as compared with WT SPNS1 (Supplemental Figure 1H). Taken together, these data suggested that P295L is functional in lysosomes but is poorly expressed.

To understand how *SPNS1* variants affect cellular lipid levels, we performed lipidomics analysis on patients and control fibroblasts from members of family A. Consistent with a defect in exporting LPC and LPE out of lysosomes, patients’ fibroblasts showed elevation of various species of LPC, LPE, and lysophospholipids containing ether bonds including plasmalogens (Figure 1I). Notably, levels of sphingosine and some of the hexosylceramide species were also elevated. The accumulation of these lysolipids were reversed by overexpression of SPNS1 (Figure 1J and Supplemental Figure 2A), supporting the conclusion that the accumulation of LPC and LPE in patients’ fibroblasts is dependent on the loss of function of *SPNS1*. Lipidomics profiling of patients’ leukocytes from family A peripheral blood revealed an increase in abundance of LPC, LPC-O/P, and LPE levels compared with levels in their parents (Supplemental Figure 2B), which is consistent with the accumulation of lysophospholipids in the fibroblast samples (Figure 1I). On the other hand, the serum levels of LPC and LPC-O/P were reduced in patients, whereas the levels of hexylceramides were elevated (Supplemental Figure 2C).

We previously showed that SPNS1 deficiency in HEK293T cells and liver results in enlarged LysoTracker-positive compartments and accumulation of the autophagy marker LC3b-II (10). Consistent with *SPNS1* loss of function, *SPNS1* patients’ fibroblasts showed increased LysoTracker-positive compartments (Figure 1, K and L), which could be due to an increase in the number and size of lysosomes. Such phenotypic changes were reversed by overexpression of WT *SPNS1* in patients’ fibroblasts (Supplemental Figure 2, D and E). However, unlike in *Spns1*-deficient mouse liver, we did not observe an increase in basal levels of LC3b-II in patients’ fibroblasts compared with levels in their age-matched controls (Supplemental Figure 2F), suggesting that the *SPNS1* mutation p.Ser416Cys, which resulted in a partial loss of function (Figure 1D), caused a less severe impairment of lysosomal function as compared with a complete absence of SPNS1 in cells (10).

### SPNS1 deficiency results in impaired neutral lipid synthesis under mTOR inhibition

**Figure 2:**
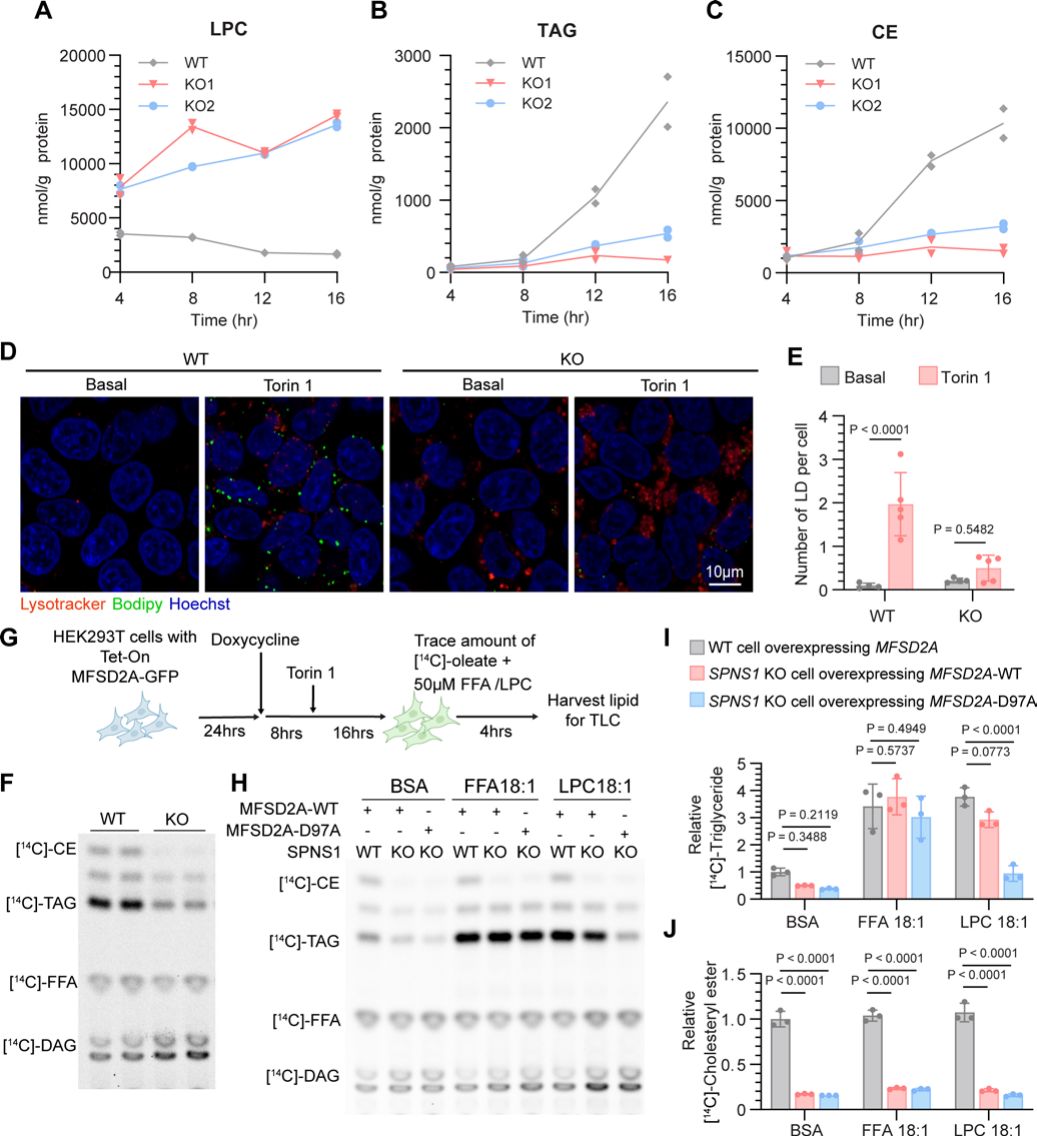
SPNS1 deficiency results in a defect in triglyceride and cholesteryl ester synthesis when mTOR activity is low. (**A**–**C**) HEK293T WT and *SPNS1*-KO (KO1, KO2) cells were treated with 250 nM Torin 1 and harvested at the indicated time points for lipidomics analysis. The concentrations of total LPC (**A**), TAG (**B**), and CE (**C**) were plotted over time. Two replicates per time point. (**D**) HEK293T WT and *SPNS1*-KO cells were treated or not with 250 nM Torin 1 for 16 hours. LDs were stained with BODIPY 493/503 (green), and lysosomes were labeled with LysoTracker (red). Hoechst staining (blue). Scale bar: 10 μm. (**E**) Quantification of the average number of LDs per cell in each field in **D**. Each data point represents 1 field, and 5 fields were scored for each condition. (**F**) HEK293T WT and *SPNS1*-KO cells were treated with 250 nM Torin 1 for 16 hours and then metabolically labeled with a trace amount of [^14^C]-oleate (FFA) for 4 hours. Formation of [^14^C]-CE, [^14^C]-TAG, and [^14^C]-DAG are shown on TLC. (**G**) Schematic representation of the radioisotope tracing study for **H**–**J**. HEK293T WT and *SPNS1*-KO cells harboring doxycycline-inducible *MFSD2A*-WT-GFP or *MFSD2A*-D97A-GFP (transport-inactive mutant) were seeded for 24 hours before treatment with 1 g/mL doxycycline for 16 hours. Torin 1 (250 nM) was added 8 hours after doxycycline treatment, and cells were incubated for another 16 hours. Cells were metabolically labeled with a trace amount of [^14^C]-oleate together with 50 μM FFA-18:1, LPC-18:1, or fatty acid–free BSA for 4 hours. Lipids were extracted from cells and analyzed by TLC. (**H**) Representative TLC results resolving [^14^C]-labeled neutral lipid species. (**I** and **J**) Quantification of [^14^C]-TAG and [^14^C]-CE bands from the TLC analysis in **H** after normalization to the protein concentration for each sample; *n* = 3. Data are presented as the mean ± SD. Statistical significance was determined by 2-way ANOVA with Šídák’s test (**E**) and 2-way ANOVA with Dunnett’s test (**I** and **J**) for each treatment group.

In an attempt to understand the pathogenesis at the cellular level of the disease caused by *SPNS1* loss-of-function variants, we further investigated the contribution of lysosomal lysophospholipid transport to cellular lipid homeostasis, an area that remains largely unexplored. Phospholipid catabolism in lysosomes has been shown to be essential for LD biogenesis during nutrient deprivation and ER stress in various cell and animal models (24–26). mTOR has a central role in the regulation of this process, serving as a nutrient sensor that when inhibited results in increased delivery of membrane phospholipids to lysosomes (21). It was shown that lysosomal fatty acids derived from the catabolism of phospholipid drive triacylglycerol (TAG) synthesis, which can either be used for β-oxidation or stored in LDs for future use (21, 27, 28). Therefore, phospholipid turnover in lysosomes and their recycling into cytosol is thought to provide an advantage to cell survival under stress. Because SPNS1 is essential for efflux of LPC from lysosomes, we hypothesized that LPC transported by SPNS1 plays a role in neutral lipid (including TAG and cholesteryl ester [CE]) storage processes when mTOR is inhibited. To test this idea, we treated WT and *SPNS1*-KO HEK293T cell lines with Torin 1, a specific mTORC1 inhibitor, and measured changes in cellular lipids over time using lipidomics analysis. As anticipated, LPC accumulated in *SPNS1*-KO cells in a time-dependent manner (Figure 2A). Strikingly, while WT cells were able to synthesize CE and TAG over time, *SPNS1*-KO cells had a greatly compromised ability to do so (Figure 2, B and C). This finding was further supported by the reduction in the number of BODIPY-positive LDs in *SPNS1*-KO cell relative to WT cells (Figure 2, D and E). Similarly, although HBSS starvation of HeLa cell induced LD formation in WT cells, LD biogenesis was reduced in *SPNS1*-KO cells (Supplemental Figure 3, A and B), indicating that the impairment of LD formation in *SPNS1*-KO cells can be generalized to other forms of nutrient deprivation.

To assay the effect of SPNS1 deficiency on the efficiency of cellular TAG and CE synthesis, we utilized radioactive fatty acid tracing to measure diacylglycerol acyltransferase (DGAT) and acyl-coenzyme A:cholesterol acyltransferase (ACAT) activity, the rate-limiting enzymes in TAG and CE synthesis, respectively. After treating cells with Torin 1 for 16 hours, a trace amount of [^14^C]-oleate was added to cells for another 4 hours, and labeled neutral lipid species were separated by thin-layer chromatography (TLC). *SPNS1*-KO cell lines showed a reduction of [^14^C]-oleate incorporated into TAG and CE (Figure 2F). The decrease in TAG synthesis could either be due to impaired DGAT enzymatic activity or reduced availability of acyl-CoA substrate. We first ruled out the former by measuring DGAT activity in an isolated membrane fraction from *SPNS1*-KO and WT cells. This DGAT activity assay indicated no significant difference in the rate of [^14^C]-TAG formation from diacylglycerol (DAG) and [^14^C]-oleoyl-CoA substrates in KO and WT cells in both basal and Torin 1-treated conditions (Supplemental Figure 3, C and D). Since LPC accumulates inside lysosomes in *SPNS1*-KO cells and LPC contains 1 covalently attached fatty acid, we reasoned that LPC that is transported out of lysosomes via SPNS1 could contribute its fatty acid for TAG synthesis. However, the existence of such crosstalk between LPC and TAG pools has not been previously demonstrated. To begin to test this concept, we added exogenous albumin-bound free fatty acid–18:1 (FFA-18:1) or LPC-18:1 to bypass the lysosome and determine whether this would rescue the defect in TAG synthesis in *SPNS1*-KO cells. To directly deliver LPC to cytosolic membranes, we constructed a doxycycline-inducible cell line expressing MFSD2A, a plasma membrane LPC transporter (29), or the transport-inactive mutant MFSD2A-D97A (30) in WT and *SPNS1*-KO cells, respectively (Figure 2G). Treatment with FFA-18:1 increased TAG synthesis in WT MFSD2A and mutant D97A cells, while LPC-18:1 treatment increased TAG synthesis only in cells expressing WT *MFSD2A* (Figure 2, H and I). Such a rescue effect of FFA-18:1 or LPC-18:1 supplementation was dose dependent (Supplemental Figure 3E). It is notable that the CE synthesis defect in *SPNS1*-KO cells was not rescued by addition of LPC-18:1 or FFA-18:1 (Figure 2, H and J), indicating that a lack of fatty acid availability in the cytosolic compartment in *SPNS1*-KO cells was not responsible for the reduced CE synthesis. Taken together, these data indicate that lysosome-derived LPC does indeed contribute to TAG synthesis and LD formation.

**Figure 3:**
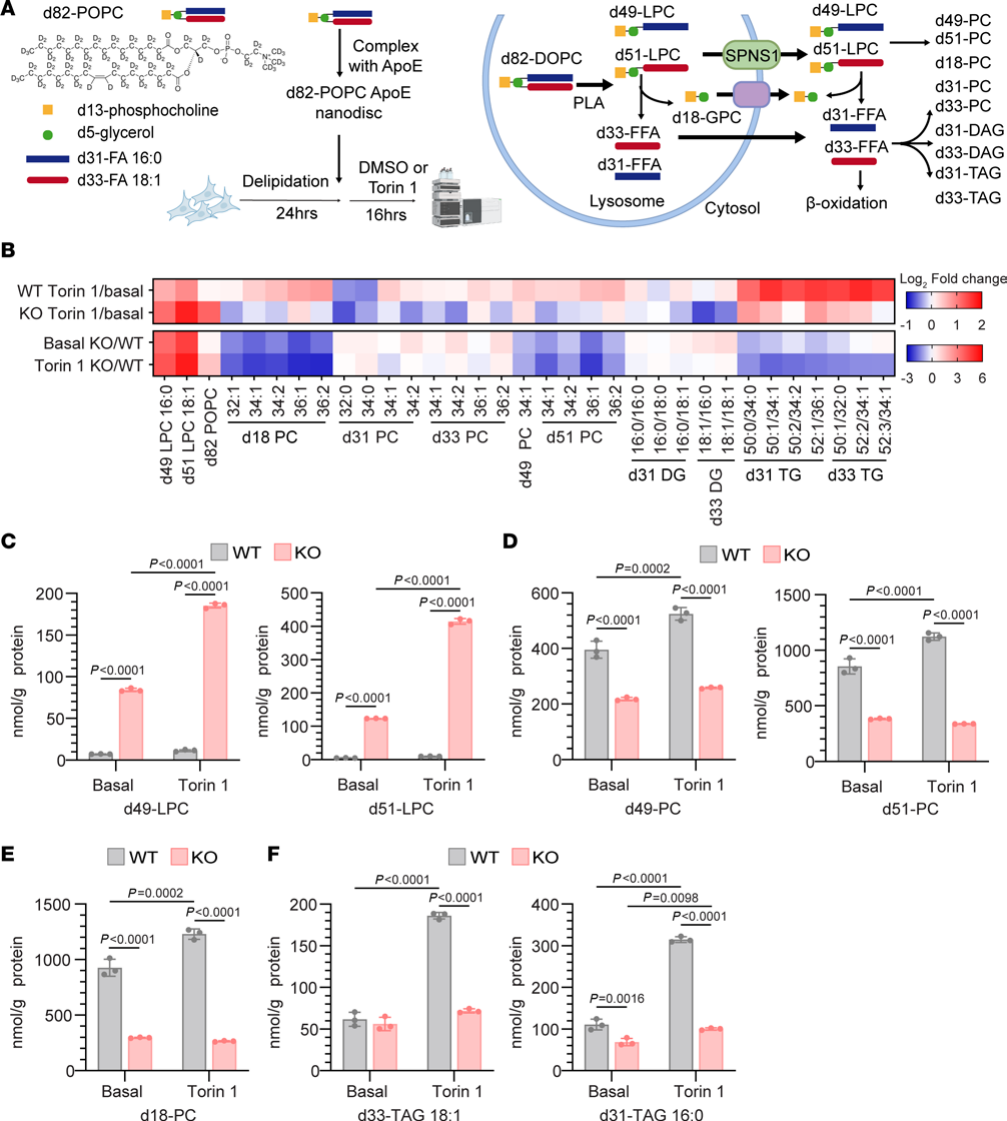
The fatty acyl moiety of LPC transported by SPNS1 contributes to triglyceride synthesis. (**A**) Structure of d82-POPC is shown together with the schematic representation of the experimental setup for the stable isotope–tracing study (**B**–**F**). The illustration on the right in **A** shows the catabolism of d82-POPC in lysosomes and potential metabolic fates of the GPC headgroup and fatty acyl tails. (**B**) Heatmap representation of the log_2_-transformed concentration of lipid species in WT cells treated with Torin 1 compared with untreated (WT Torin 1/basal), *SPNS1*-KO cells treated with Torin 1 compared with untreated (KO Torin 1/basal), KO cells compared with WT cells, both under basal conditions (Basal KO/WT), and KO cells compared with WT cells both with Torin 1 treatment (Torin 1 KO/WT). *n* = 3 replicates. *P* values were calculated using 2-tailed, unpaired Student’s *t* tests and are presented in the Supplemental Data. (**C**–**F**) Concentration of per-deuterated LPC (d49-LPC, d51-LPC) (**C**), PC having labeled LPC (d49-PC, d51-PC) (**D**), PC having labeled GPC (d18-PC) (**E**), and TAG having labeled fatty acid (d31-TAG, d33-TAG) (**F**). *n* = 3 replicates. Data are presented as the mean ± SD. Statistical significance was determined by 2-way ANOVA with Tukey’s test.

To provide an independent line of evidence that lysosome-derived LPC quantitatively contributes to supplying fatty acid for TAG synthesis during mTOR inactivation, we conducted a stable isotope-tracing study to follow the metabolic fate of PC delivered to the lysosome. Similar to our previously published experimental method, we complexed per-deuterated d82–1-palmitoyl-2-oleoyl-sn-glycero-3-phosphocholine (d82-POPC) with apoE to form nanodiscs that are delivered to lysosomes thorough low-density lipoprotein receptor–mediated (LDLR- mediated) endocytosis (10). The use of d82-POPC allowed us to trace all components (i.e., fatty acids, glycerol backbone, and choline headgroup) of this PC into cellular lipids. Inside the lysosome, we anticipated that d82-POPC would be hydrolyzed to d49-LPC16:0 and d33-FFA18:1 by lysosomal PLA2 and to d51-LPC18:1 and d31-FFA16:0 by lysosomal PLA1. The LPC transported out of lysosomes via SPNS1 can be used directly for phosphatidylcholine synthesis or hydrolyzed further to release fatty acid (Figure 3A). Together with the fatty acid released directly from PC hydrolysis to LPC, these fatty acids could be utilized for phospholipid remodeling, DAG and TAG synthesis, and β-oxidation (Figure 3A). As such, we quantified PC, TAG, and DAG species that contain 1 or more of the d33-18:1 fatty acyl chain, the d31-16:0 fatty acyl chain, the d5-glycerol backbone, or d13-phosphocholine (Figure 3A).

Consistent with SPNS1 being a lysosomal LPC transporter, we found that *SPNS1*-KO cells accumulated d49-LPC16:0 and d51-LPC18:1 (Figure 3, B and C). The higher d51-LPC18:1 versus d49-LPC16:0 levels suggested that PLA1 activity might be more predominant than PLA2 activity in HEK293T cells. Consistent with our previous study that traced the fate of d9-choline in d9-1,2-dioleoyl-sn-glycero-3-phosphocholine (DOPC), *SPNS1*-KO cells had reduced recycling of the glycerophosphocholine (GPC) headgroup into to PC pools (Figure 3B). We observed a 50% reduction of d51-LPC-18:1 or d49-LPC-16:0 containing PC generated from direct re-acylation of lysosome-derived LPC by 12) lysophosphatidylcholine acyltransferase activity in *SPNS1*-KO cells (Figure 3D). Since d51-PC and d49-PC can undergo acyl chain remodeling through Land’s cycle, we also observed a reduction in PC species containing only the deuterated GPC headgroup (d18-PC) in *SPNS1*-KO cells (Figure 3E). Moreover, such LPC accumulation and deficiency of GPC headgroup salvage was exacerbated by Torin 1 treatment (Figure 3, B–E), as mTOR inhibition can increase both endocytic delivery of lipid and lysosomal hydrolase activity (31, 32). These findings suggested that the SPNS1-mediated phospholipid salvage pathway is quantitatively important for maintaining cellular phospholipid homeostasis, especially when mTOR activity is low.

We next quantified the fate of d31 and d33 fatty acyl groups in *SPNS1*-KO and WT cells. WT cells increased the incorporation of d31-FA-16:0 and d33-FA-18:1 into TAG upon mTOR inhibition by 3-fold (Figure 3, B and F), confirming the ability of cells to utilize fatty acids derived from lysosomal phospholipid catabolism. In contrast, *SPNS1*-KO cells had only slightly increased incorporation of d31-FA-16:0 and d33-FA-18:1 into TAG after Torin 1 treatment (Figure 3B), resulting in a more than 50% reduction in TAG containing d33-FA-18:1 or d31-FA-16:0 when compared with WT cells under mTOR inhibition (Figure 3F). This finding is consistent with the 50% reduction in [^14^C]-oleate labeling of TAG in our radioisotope tracing study (Figure 2I). On the other hand, d31-FA-16:0 and d33-FA-18:1 incorporation into PC (d31-PC and d33-PC) and DAG (d31-DAG, D33-DAG) was minimally affected by the absence of SPNS1 (Supplemental Figure 4, A and B), suggesting a preferential use of lysosome-derived fatty acids to maintain PC and DAG levels. We noticed that in WT cells without mTOR inhibition, only 2.5% of d33-FA-18:1 and 4.8% of d31-FA-16:0 derived from d82-POPC was channeled for TAG synthesis, while this proportion increased to 6% and 11%, respectively, after Torin 1 treatment (Supplemental Figure 4C). In contrast, we did not see such an increase of these labeled fatty acids in TAG in *SPNS1*-KO cells (Supplemental Figure 4C). These findings support the existence of an adaptive shift in the fate of fatty acids derived from lysosomal LPC in response to changes in mTOR activity and indicate that this process is dependent on SPNS1-mediated LPC transport.

### Deficiency of SPNS1 results in defective lysosomal cholesterol efflux

**Figure 4:**
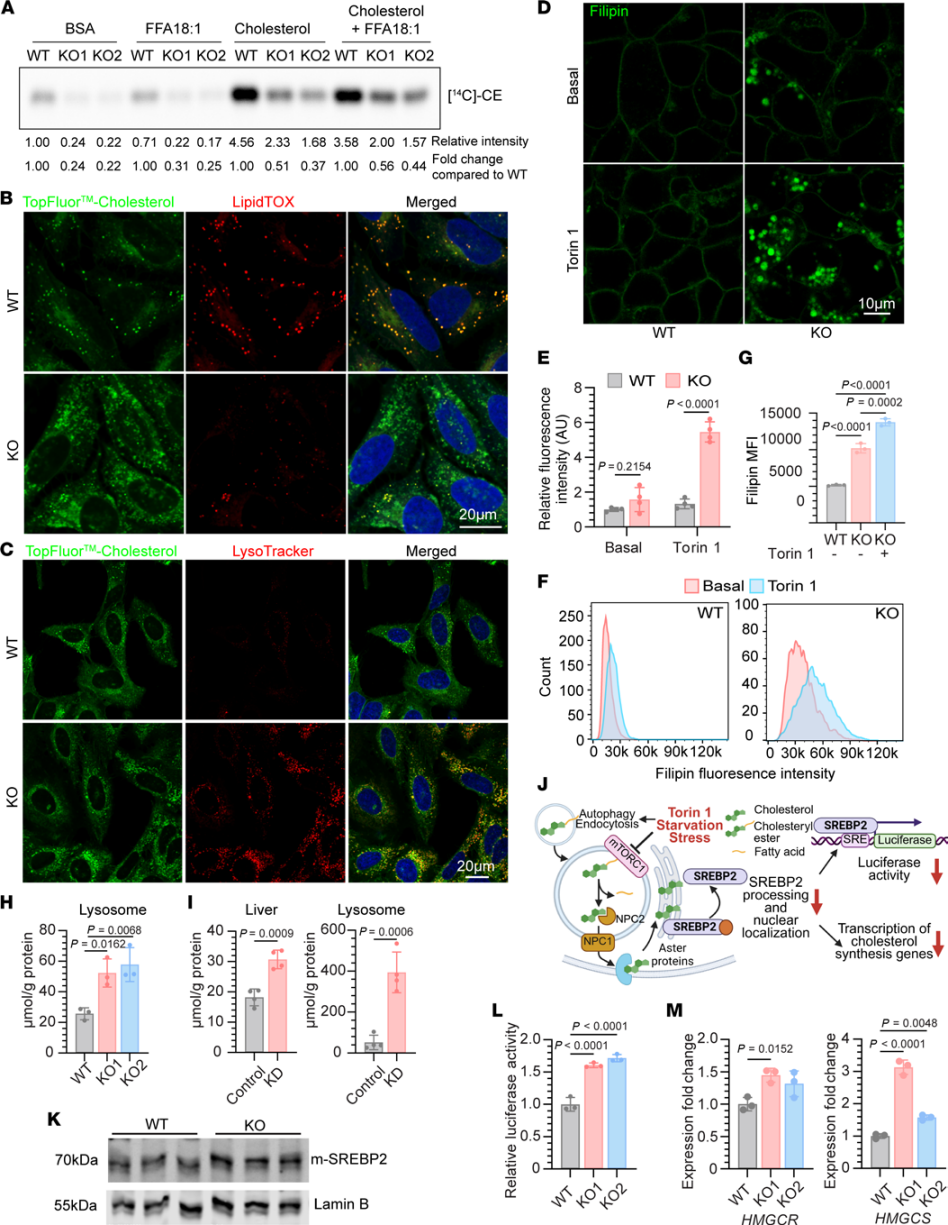
*SPNS1*-KO cells have defective lysosomal cholesterol egress. (**A**) Quantification of CE synthesis in HEK293T WT and *SPNS1*-KO cells. After 16 hours of Torin 1 treatment, a trace amount of [^14^C]-oleate with fatty acid–free BSA, 20 μM FFA-18:1, 35.7 μM cholesterol-MCD complex, or 20 μM FFA-18:1 and the 35.7 M cholesterol-MCD complex were added to cells for 4 hours. The fold change of [^14^C]-CE band intensity relative to WT (BSA) or WT for each treatment group was calculated. (**B**) *SPNS1*-KO and WT HeLa cells were labeled with TopFluor Cholesterol (green) for 24 hours, followed by 16 hours of treatment with Torin 1. Fixed cells were stained with LipidTOX (red) for LDs. Scale bar: 20 μm. (**C**) Live cell imaging of HeLa *SPNS1*-KO and WT cells pulse labeled with TopFluor Cholesterol (green) complexed with MCD, followed by 16 hours of treatment with Torin 1. Hoechst staining (blue). Scale bar: 20 μm. (**D**) Filipin staining of HEK293T WT and *SPNS1*-KO cells treated with or without Torin 1. Scale bar: 10 μm. (**E**) Quantification of filipin staining fluorescence intensity in each field in **D**. Four different fields were scored for each condition. (**F**) Representative flow cytometry profile of filipin intensity of HEK293T WT and *SPNS1*-KO cells treated or not with Torin 1. Plasma membrane cholesterol was depleted using MCD before filipin staining. (**G**) MFI of filipin staining measured by flow cytometry in **F**. *n* = 3 replicates. (**H**) Concentration of lysosomal cholesterol from HEK293T WT and *SPNS1*-KO cell. *n* = 3 replicates. (**I**) Cholesterol levels in lysosomes and liver tissue from mice injected with adeno-associated virus serotype 8 (AAV8) carrying an shRNA targeting *Spns1* (KD) or nontargeting shRNA (control). *n* = 4 mice per group. (**J**) Schematic illustration of how mTOR regulates lysosomal cholesterol egress to suppress activation of SREBP2. Illustration was created with BioRender. (**K**–**M**) HEK293T WT and *SPNS1*-KO cells were grown in delipidated media for 16 hours. FBS (10%) and Torin 1 were added for another 8 hours before harvesting the cells. (**K**) Immunoblotting for mature SREBP2 (m-SREBP2) and lamin B in nuclear fractions. (**L**) Luciferase reporter assay for SREBP transcriptional activity. (**M**) mRNA expression of select SREBP2 target genes. *n* = 3 replicates. Data are presented as the mean ± SD. Statistical significance was determined by 2-way ANOVA with Šídák’s test for (**E**), 1-way ANOVA with Tukey’s test (**G**), 1-way ANOVA with Dunnett’s test (**H**, **L**, and **M**), and 2-tailed, unpaired Student’s *t* test (**I**).

Although the defect in TAG synthesis in *SPNS1*-KO cells was rescued by supplementing cells with fatty acids or LPC, the defect in CE synthesis was not (Figure 2, H–J), suggesting that *SPNS1*-KO cells had a deficiency in ER cholesterol or compromised ACAT activity. Cells acquire cholesterol through de novo synthesis and endocytosis of lipoproteins. NPC1 and NPC2 mediate the egress of LDL-derived cholesterol out of the lysosome (1, 2), and the lysosome-derived cholesterol first travels to the plasma membrane before trafficking to the ER, a process mediated by Aster proteins (33–35). We first examined if ACAT activity was compromised in *SPNS1*-KO cells by testing whether enriching plasma membrane cholesterol using the methyl-β cyclodextrin-cholesterol (MCD) complex could rescue the CE synthesis defect in *SPNS1*-KO cells. Radioisotope tracing showed that both *SPNS1*-KO and WT cells were able to increase the incorporation of [^14^C]-oleate into [^14^C]-CE upon cholesterol supplementation, indicating that ACAT was functional in esterifying plasma membrane–derived cholesterol in *SPNS1*-KO cells (Figure 4A). While KO cells synthesized 75% less [^14^C]-CE than did WT cells under basal conditions, supplementation with cholesterol alone or with both cholesterol and fatty acids only partially normalized the difference in CE synthesis, as KO cells synthesized 50% less [^14^C]-CE than did WT cells under these conditions (Figure 4A). This could be due to a defect in either lysosomal egress or plasma membrane–to–ER trafficking of cholesterol. The former would lead to lysosomal accumulation of cholesterol, whereas the latter would lead to sequestration of cholesterol on the plasma membrane. Therefore, we used TopFluor Cholesterol, a fluorescent cholesterol analog that can be esterified by fatty acids, to metabolically labeled cellular cholesterol and visualize its subcellular localization after Torin 1 treatment. In WT cells, TopFluor Cholesterol had a diffuse ER and PM staining pattern that colocalized with LipidTOX-positive LDs after Torin 1 treatment, indicating that TopFluor Cholesterol was channeled to cytosolic LDs (Figure 4B and Supplemental Figure 5A). In contrast, *SPNS1*-KO cells had a punctate pattern of TopFluor Cholesterol that colocalized with LysoTracker (Figure 4C), demonstrating that cholesterol was delivered to cells using MβCD and accumulated in lysosomes of *SPNS1*-KO cells. These findings likely explain the partial rescue of CE synthesis in *SPNS1*-KO cells treated with exogenously added cholesterol delivered by MβCD (Figure 4A), as some of the cholesterols are sequestered in the lysosome and not available for acylation by ACAT.

To further examine the cellular distribution of endogenous cholesterol, cells were stained with filipin, a fluorescent dye that specifically binds to unesterified cholesterol. We observed an intracellular punctate staining pattern in *SPNS1*-KO cells, whereas WT cells exhibited primarily plasma membrane staining (Figure 4D). Such accumulation became more profound in Torin 1–treated *SPNS1*-KO cells (Figure 4, D and E) and was also observed in other cell types with SPNS1 deficiency, namely HeLa and HuH-7 cells (Supplemental Figure 5, B and C). As expected, there was a corresponding increase in cellular LPC levels in *SPNS1*-KO HeLa or HuH-7 cells that was exacerbated with Torin 1 treatment (Supplemental Figure 5D). We also developed a flow cytometry method to quantify intracellular filipin staining intensity. As more than 90% of free cholesterol is estimated to reside in the plasma membrane (36, 37), we used a short-term treatment of cells with MCD to deplete plasma membrane cholesterol before staining with filipin (38), so that lysosomal filipin staining would be the primary signal being measured. We verified that our treatment was effective in reducing plasma membrane filipin staining without changing the punctate staining pattern (Supplemental Figure 5E). The reduction in total filipin fluorescence after MCD treatment was measured by flow cytometry (Supplemental Figure 5F). Using this method, we verified that *SPNS1*-KO cells had increased intracellular cholesterol levels relative to WT cells, even in the basal state, with Torin 1 treatment further increasing filipin staining in *SPNS1*-KO cells (Figure 4, F and G).

To confirm lysosomal cholesterol accumulation in *SPNS1*-KO cells, we performed lipidomics analysis on isolated lysosomes and observed a 2-fold increase in lysosomal unesterified cholesterol (Figure 4H). Similar results were obtained when we metabolically labeled cells with [^14^C]-cholesterol and subjected the extracted lipids from isolated lysosomes to TLC (Supplemental Figure 5, G and H). Finally, we reanalyzed the lipidomics data from our previous study of liver-specific *Spns1*-knockdown mice (10) and found a large increase in cholesterol in both liver and isolated lysosomes from *Spns1*-knockdown mouse liver (Figure 4I), indicating that such a disturbance in lysosomal and cellular cholesterol homeostasis also occurred at the organ level.

To more specifically determine whether *SPNS1*-KO cells have defective lysosomal cholesterol egress, cells were delipidated overnight followed by LDL loading, which would deliver cholesterol and cholesteryl ester directly to lysosomes. Cells were either labeled with [^14^C]-oleate to quantify [^14^C]-CE synthesis or stained with filipin to quantify endolysosomal cholesterol. With this approach, we observed an approximately 20% reduction in the incorporation of [^14^C]-oleate into [^14^C]-CE (Supplemental Figure 6A) as well as a significant increase in the intracellular punctate distribution of filipin staining in *SPNS1*-KO cells that was exacerbated by Torin 1 treatment (Supplemental Figure 6, B and C). Collectively, these findings suggest that cholesterol accumulates in the lysosomes of *SPNS1*-KO cells due to reduced lysosomal cholesterol egress.

Given that cellular cholesterol is sequestered in lysosomes in *SPNS1*-KO cells, we wondered whether that would result in a deficiency of ER and plasma membrane cholesterol, especially in delipidated conditions when there are no exogenous sources of cholesterol. It was reported that mTORC1 activates SREBP2 by suppressing cholesterol trafficking from lysosomes to the ER, while mTORC1 inhibition has the opposite effect by activating lysosomal cholesterol egress, resulting in suppression of SREBP2 processing (39). We hypothesized that if *SPNS1*-KO cells have reduced lysosomal cholesterol egress, then there should be a blunting of SREBP2 suppression under conditions in which cholesterol egress is enhanced (Figure 4J). To examine this possibility, we cultured WT and *SPNS1*-KO cells in medium supplemented with delipidated FBS overnight to upregulate SREBP2 activity and then added back FBS and Torin 1 to stimulate the egress of cholesterol from the lysosome to the ER. Under these experimental conditions, *SPNS1*-KO cells had an increased mature form of SREBP2 (nuclear SREBP2) relative to WT cells (Figure 4K). To further validate this observation, we used a classic sterol response element (SRE) luciferase reporter assay to quantify the transcriptional activity of SREBP. Consistent with increased SREBP2 processing, we found that SREBP activity in *SPNS1*-KO was elevated relative to WT cells (Figure 4L). We further confirmed this finding with reverse transcription quantitative PCR (RT-qPCR), which showed upregulation of several classical SREBP2 target genes, namely *HMGCR* and *HMGCS*, in *SPNS1*-KO cells relative to WT cells (Figure 4M). It is notable that *SPNS1*-KO cells exposed to delipidated media already had elevated SREBP activity relative to WT cells (Supplemental Figure 6D), further suggesting that the *SPNS1*-KO cells had reduced ER cholesterol. Together, these findings support the conclusion that SPNS1 deficiency results in a defect in lysosomal cholesterol egress.

### The accumulation of ether-LPC and ether-LPE impedes lysosomal cholesterol egress

**Figure 5:**
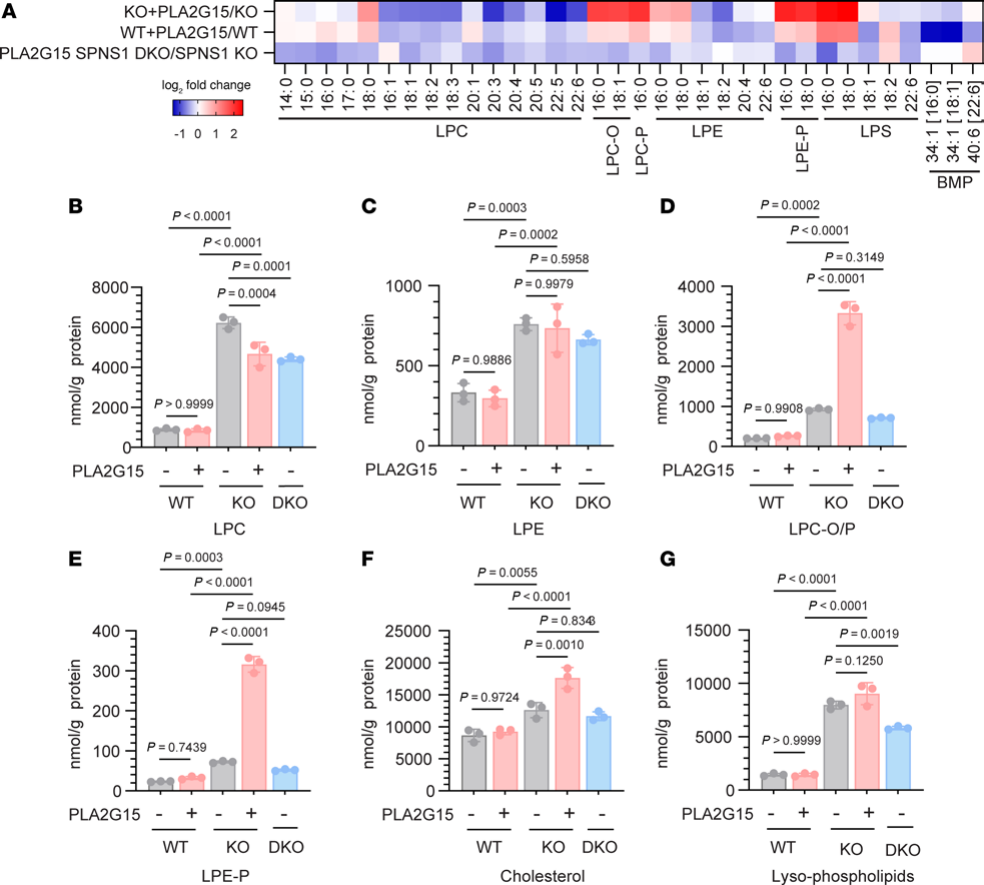
PLA2G15 enzyme supplementation increases lysosomal cholesterol. HEK293T WT and *SPNS1*-KO cells were supplemented with 3 g/mL purified PLA2G15 for 48 hours, and plasma membrane cholesterol was depleted using MCD before harvesting cells for lipidomics analysis. (**A**) Heatmap representation of log_2_ fold change of lipid concentrations in *SPNS1*-KO and WT cells with or without PLA2G15 supplementation, and *PLA2G15 SPNS1* double-KO (DKO) relative to *SPNS*1-KO cells. *n* = 3 replicates. *P* values were calculated using 2-tailed unpaired Student’s *t* tests and are presented in the Supplemental Data. (**B**–**G**) Cellular concentrations of LPC, LPE, LPC-O/P, LPE-P, and cholesterol and the sum of all lysophospholipids transported by SPNS1 (LPC, LPE, LPC-O/P, LPE-P). *n* = 3 replicates. Data are presented as the mean ± SD. Statistical significance was determined by 1way ANOVA with Šídák’s test.

As the accumulation of LPC and cholesterol in lysosomes of *SPNS1*-KO cells was further increased by mTOR inhibition (Figure 2A, Figure 4D, and Supplemental Figure 5, B–D), we sought to test whether there exists a causal relationship between lysosomal LPC levels and cholesterol accumulation. We utilized PLA2G15, a lysosomal phospholipase A2 (PLA2) that also has phospholipase B (PLB) activity (8), to alter lysosomal LPC levels. PLA2G15 is reported to hydrolyze PC, PE, PG, PS, their corresponding lysophospholipids, and bis(monoacylglycerol)phosphate (BMP) in in vitro enzyme activity assays (8, 40). Therefore, PLA2G15 could either decrease or increase lysosomal LPC levels depending on whether its PLA2 or PLB activity is predominant. We purified His-tagged PLA2G15 to homogeneity (Supplemental Figure 7, A and B) and supplemented the WT and *SPNS1*-KO cells with PLA2G15 and then performed lipidomics analysis. Consistent with previous reports (8), treating cells with purified PLA2G15 resulted in cellular uptake of the enzyme (Supplemental Figure 7C). Lipidomics analysis revealed that LPC, LPE, and LPS with mono- or polyunsaturated fatty acids were decreased with PLA2G15 treatment, while LPC, LPE, and LPS containing saturated fatty acids remained unchanged or were slightly elevated (Figure 5A). These changes are consistent with PLA2G15 having PLA2 activity that hydrolyzes the mono- and polyunsaturated fatty acids, which are usually esterified at the sn-2 position on the glycerol backbone. Interestingly, while the total LPC and LPE was either reduced or unchanged (Figure 5, A–C), the levels of LPC and LPE containing ether or vinyl-ether bonds (LPC-O/P and LPE-P) were greatly elevated upon supplementation of PLA2G15 in *SPNS1*-KO cells but not in WT cells (Figure 5, A, D, and E). This suggests that PLA2G15 is specific to the phospholipid ester bond and does not possess etherase activity. Indeed, purified PLA2G15 was able to digest PC, LPC, and plasmalogen containing PC (PC-P) to release FFAs but was unable to hydrolyze LPC-P (Supplemental Figure 7D). In an attempt to reduce lysosomal LPC-O/P and LPC-P levels, we knocked out *PLA2G15* in *SPNS1*-KO cells. However, double-KO cells showed no significant changes in either LPC/LPE or LPC-O/P and LPE-P levels (Figure 5, A–E), indicating the existence of other phospholipases in lysosomes that can generate lysophospholipids in the absence of PLA2G15.

To determine the effect of reduced lysosomal LPC/LPE with increased LPC-O/P and LPE-P on lysosomal cholesterol, we quantified filipin staining by flow cytometric analysis. We found that intracellular filipin staining, a marker for lysosomal cholesterol, was unchanged in WT cells with PLA2G15 supplementation but was significantly elevated in *SPNS1*-KO cells in both basal and Torin 1–treated conditions (Supplemental Figure 7E), a finding that was confirmed by lipidomics analysis (Figure 5F). The accumulation of cholesterol in *SPNS1*-KO cells treated with PLA2G15 was unlikely, given the reduction of BMP, a substrate of PLA2G15, because WT cells treated with PLA2G15 had a larger drop in BMP levels than did *SPNS1*-KO cells without changes in cholesterol levels (Figure 5A). Importantly, the total amount of lysophospholipids (LPC, LPC-O/P, LPE, LPE-P) was unchanged with PLA2G15 supplementation in KO cells (Figure 5G). Therefore, it is likely that the accumulation of LPC-O/P and LPE-P, but not LPC and LPE, was causative for the lysosomal cholesterol egress defect in *SPNS1*-KO cells.

### Patients’ fibroblasts show a defect in cholesterol egress and neutral lipid synthesis under mTOR inhibition

**Figure 6:**
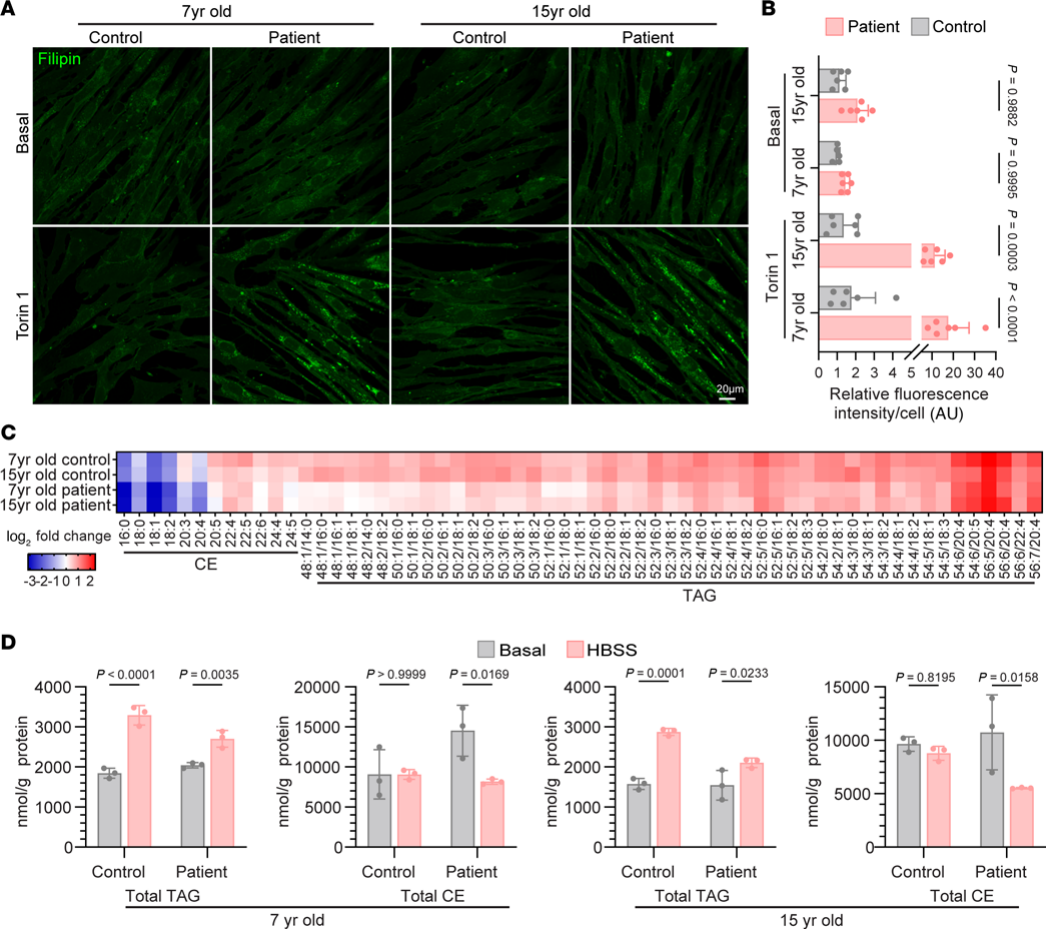
Fibroblasts from *SPNS1* patients have increased cholesterol and reduced neutral lipid synthesis under inhibition of mTOR. (**A**) Filipin staining of fibroblasts from patients and age-matched controls with or without 16 hours of 250 nM Torin 1 treatment. Scale bar: 20 μm. (**B**) Quantification of filipin staining fluorescence intensity per cell in each field. Each data point represents 1 field, and 5 different fields were scored. (**C**) Heatmap representation of log_2_ fold change of neutral lipids species (CE and TAG) of each fibroblast cell line after 6 hours of starvation in HBSS. *P* values were calculated using 2-tailed, unpaired Student’s *t* tests and are presented in the Supplemental Data. (**D**) Concentration of total TAG and CE in patients’ fibroblasts and age-matched control fibroblasts before and after HBSS treatment. *n* = 3 replicates. Data are presented as the mean ± SD. Statistical significance was determined by 2-way ANOVA with Šídák’s test (**B** and **D**).

We next set out to test whether patients’ fibroblasts with mutant *SPNS1* exhibited disturbances in cellular lipid homeostasis similar to those found in *SPNS1*-KO HEK293T cells. We first confirmed by filipin staining that patients’ fibroblasts, but not their age-matched controls, accumulated cholesterol in lysosomes upon mTOR inhibition (Figure 6, A and B). We similarly used MCD to deplete plasma membrane cholesterol and measured cellular lipid levels by lipidomics analysis. Torin 1 treatment resulted in significantly increased levels of LPC and LPC-O in patients’ fibroblasts and, to a much lower extent, in controls, as well as a slight increase in cellular cholesterol levels (Supplemental Figure 8). We next looked at the rate of neutral lipid synthesis upon placing cells in HBSS media to induce amino acid starvation, a condition that reduces mTOR activity. Both control and patients’ fibroblasts were able to increase the synthesis of TAG and some classes of CE upon amino acid starvation, but the extent of the increase was lesser in patients’ fibroblasts (Figure 6, C and D). These data are consistent with our findings that LPC transported out of lysosomes by SPNS1 contributed fatty acids for TAG in LD biogenesis.

## Discussion

In this study, we identified members of 2 families with biallelic nonsynonymous variants in *SPNS1* that inactivate transporter function. The patients primarily presented with progressive muscle weakness, elevated creatinine, persistent elevation of liver transaminase and liver injury. The clinical presentation of the patients was also similar to phenotypes observed in mouse models of *Spns1* deficiency (10, 11, 15). We confirmed that the disease-causing mutations of *SPNS1* resulted in protein with reduced transport function or poorly expressed protein. We carried out an in-depth characterization of lipidomic changes and cellular phenotypes using patients’ fibroblasts from family A with heterozygous mutations and confirmed elevation of LPC, LPE, as well as other sphingolipid metabolites in the patients’ fibroblasts. This represents a lysosomal storage disease caused by *SPNS1* variants and defines a gene-disease association. It is notable that the clinical phenotypes of our patients differed from the phenotypes of a previously reported single pedigree of 3 patients with a homozygous variant (p.Pro295Leu) of *SPNS1* that presented mainly with neurological symptoms such as cerebellar defects and neurodevelopment delay, without liver or muscle involvement (11). We further characterized this variant and found that P295L was poorly expressed but fully complemented elevated LPCs in *SPNS1*-KO cells. It would be important to determine whether the p.Pro295Leu variant causes LPC accumulation in cells in order to confirm causation of disease in these patents. Nevertheless, it is possible that variants in *SPNS1* could lead to a spectrum of disease.

In the attempt to understand the pathogenesis of this newly characterized lysosomal storage disease, we discovered additional roles of SPNS1 in maintaining cellular lipid homeostasis other than phospholipid salvage. While lysosomal choline recycling might be important for cancer cell survival in choline-deficient conditions (12), essential deficiency of choline is rare (13). *SPNS1*-KO cells and animals do not have PC deficiency (10, 11). As such, it is unlikely that the disease of patients with an *SPNS1* variants is caused by inefficient choline salvage from lysosomes. Therefore, we sought to understand the full metabolic fate of LPC transported out of lysosomes. While it has been shown that phospholipid synthesis is important for LD expansion (41) and that addition of exogenous LPC to cells can induce LD formation (42), direct evidence for crosstalk between phospholipid catabolism and TAG synthesis was lacking. We provided multiple lines of evidence that lysosomal LPC transported by SPNS1 quantitatively contributed its fatty acid for TAG synthesis when mTOR activity was reduced, linking the catabolism of lysosomal LPC to TAG synthesis and storage in cytosolic LDs. The identity of the enzymes that mediate the metabolism of LPC for TAG synthesis is not known. More recently, TMEM68/DISEL has been identified as an alternative DGAT that generates TAG by potentially utilizing membrane phospholipids as the fatty acyl donor (43, 44). It remains to be explored whether TMEM68 can directly use lysosome-derived LPC or PC derived from re-acylation of lysosome-derived LPC as a substrate for TAG synthesis.

One of the more unexpected findings from our study was that SPNS1 deficiency led to a reduction in lysosomal cholesterol egress that was due in part to accumulation of lysosomal ether-LPC/LPE. Our experimental use of PLA2G15, a known lysosomal phospholipase, allowed us to make a compositional change in the lysosomal lipidome without altering the total amount of lysophospholipids. This method helped to pinpoint alkyl and vinyl ether LPC (LPC-O/P) and LPE (LPE-P) as likely causative lipid species leading to reduced lysosomal cholesterol egress in *SPNS1*-KO cells. This effect was SPNS1 dependent because PLA2G15 supplementation in WT cells did not increase the levels of LPC-O/P or LPE-P, which are substrates for SPNS1 transport (10). Although it is possible that some of the lysosomal LPC and LPE are hydrolyzed further to GPC and glycerophosphorylethanolamine (GPE) by PLA2G15 (8), our findings indicate that LPC-O/P and LPE-P are entirely reliant on SPNS1 to exit the lysosome. Indeed, to our knowledge, there is no reported lysoplasmalogenase activity in lysosomes. So far, the only identified lysoplasmalogenase is TMEM86A/B, which localizes to the ER (45, 46). Therefore, a potential evolutionary role of SPNS1 could be for lysosomal LPC-O/P and LPE-P transport and salvage. How LPC-O/P and LPE-P accumulation contribute to cholesterol accumulation is unknown. Interestingly, deficiency of cellular plasmalogen was shown to be associated with defects in cellular cholesterol trafficking and HDL-mediated cholesterol efflux (47, 48). In fact, TMEM86A was reported to be regulated by the sterol-controlled liver X receptor (49), further suggesting an association between cellular plasmalogen levels and cholesterol trafficking. While LPC itself has been reported to form complexes with cholesterol at a 1:1 molar ratio in in vitro systems (50), the possibility remains to be tested that physical interactions between LPC-O/P and LPE-P and cholesterol, and potentially other accumulated lipids such as sphingosine and ceramide, impede cholesterol movement inside the lysosome. Alternatively, LPC-O/P and LPE-P may compete or affect the binding of cholesterol to NPC2 and NPC1. Edelfosine, a synthetic alkyl-lysophospholipid structurally similar to LPC-O, was shown to inhibit cholesterol binding to purified NPC2 and the N-terminus of NPC1 (51, 52). Reassuringly, when we performed data mining on an existing CRISPR screen for regulators of cellular cholesterol by treating cells with a NPC1 inhibitor (53), *SPNS1* emerged as a positive regulator of lysosomal cholesterol egress.

How might these lysosomal and cellular lipid changes contribute to the disease pathogenesis in *SPNS1* loss of function is less clear. Although we observed no deficiency of PC or PE species in patients’ fibroblasts, we detected a decrease in plasmalogen PC and PE levels with an associated increase in LPC-O/P and LPE-P (Figure 1I). It is tempting to speculate that lysosomal transport of LPC-O/P and LPE-P by SPNS1 forms an ether-phospholipid salvage pathway that could be physiologically important for maintaining cellular plasmalogens. Plasmalogens have diverse functions ranging from serving as a reservoir for signaling molecules, modulating membrane properties to being an antioxidant (54–56). Defective lysosomal cholesterol egress associated with lysosomal accumulation of LPC-O/P and LPE-P in SPNS1 deficiency is reminiscent of NPC1/2 disease and could be a contributing factor in causing disease in patients with the *SPNS1* variants. Other possible causes of disease in patients with the *SPNS1* variant could relate directly to a reduced ability of cells to adapt to less amino acid availability, either through a lower ability to store energy as TAG in LDs and/or defective autophagy. LPC and LPE have detergent-like properties that could lead to lysosomal dysfunction that could be exacerbated during nutrient starvation, when autophagy is important for clearance of damaged cellular organelles (57). A general defect in autophagy could lead to gradual accumulation of damaged organelles such as mitochondria, which could be toxic to cell viability (58). Although LC3b-II levels were unchanged in patients’ fibroblasts, we cannot rule out the possibility that autophagy was not affected in the tissues of patients with the *SPNS1* variants.

Taken together, our study reports a lysosomal storage disease resulting from inactivating mutations in *SPNS1* that revealed crosstalk between lysosomal lysophospholipid transport with the regulation of cellular cholesterol and TAG homeostasis.

## Methods

Additional details can be found in Supplemental Methods.

### Sex as a biological variable.

Our study examined 2 families with rare biallelic variants in *SPNS1*. Both sexes were examined, with 3 affected males, 1 female heterozygous carrier, and 1 male heterozygous carrier.

### Statistics.

Statistical analysis was performed in GraphPad Prism (GraphPad Software) using a 2-sided, unpaired Student’s *t* test and 1-way and 2-way ANOVA with Dunnett’s or Turkey’s test as indicated. The number of independent experiments for each figure are indicated in the figures and legends. All experiments were carried out at least in triplicate. Exact *P* values for the lipidomics analysis are reported in the Supplemental Data Set 1. A *P* value of less than 0.05 was considered significant. All data in the figures are presented as the mean ± SD.

### Study approval.

The study on family A was approved by the Regional Ethical Review Board in Stockholm, Sweden (ethics permit number 2019-04746) in accordance with the Declaration of Helsinki. Informed consent was obtained from the legal guardians. The study on family B was approved by the local ethics committee of the Technical University Munich (5360/121 S). Both parents gave written informed consent, and the study was conducted in compliance with the Declaration of Helsinki.

### Data availability.

The patients’ genomic data cannot be deposited in a public repository due to legal and ethics restrictions under Swedish, German, and EU regulations, including General Data Protection Regulation. Variant-level data (gene, HGVS, zygosity, inheritance, and population frequency) are provided in the manuscript. Further details and patient data are available with a transfer agreement upon request to the corresponding author. Values for all data points in the graphs are reported in the Supporting Data Values file. Lipidomics data are provided in Supplemental Data Set 1.

## Author contributions

MH and DLS conceptualized the study. MH, FT, and DLS designed the study methodology. MH performed experiments. ME, HM, MCVM, VA, M Durbeei, ADV, TS, AN, MW, MCL, and JH performed genetic and clinical analyses. M Ding, MC, and FT performed data acquisition and analysis. DLS supervised the study. MH and DLS wrote the original draft of the manuscript. All authors reviewed the manuscript and provided input. DLS, FT, CFC, AN, TS, M Durbeei, and MCL were responsible for funding acquisition.

## Supplementary Material

Supplemental data

Supplemental data set 1

Unedited blot and gel images

Supporting data values

## Version 1 07/03/2025

In-Press Preview

## Version 2 09/02/2025

Electronic publication
