# Supplementary material for: SPNS1 variants cause multiorgan disease and implicate lysophospholipid transport as critical for mTOR-regulated lipid homeostasis
Source: J Clin Invest. 2025 Jul 3;135(17):e193099. doi: 10.1172/JCI193099 (PMC12404768; doi:10.1172/JCI193099)
Supplement: Supplemental data [file jci-135-193099-s041.pdf]

Supplemental Figures

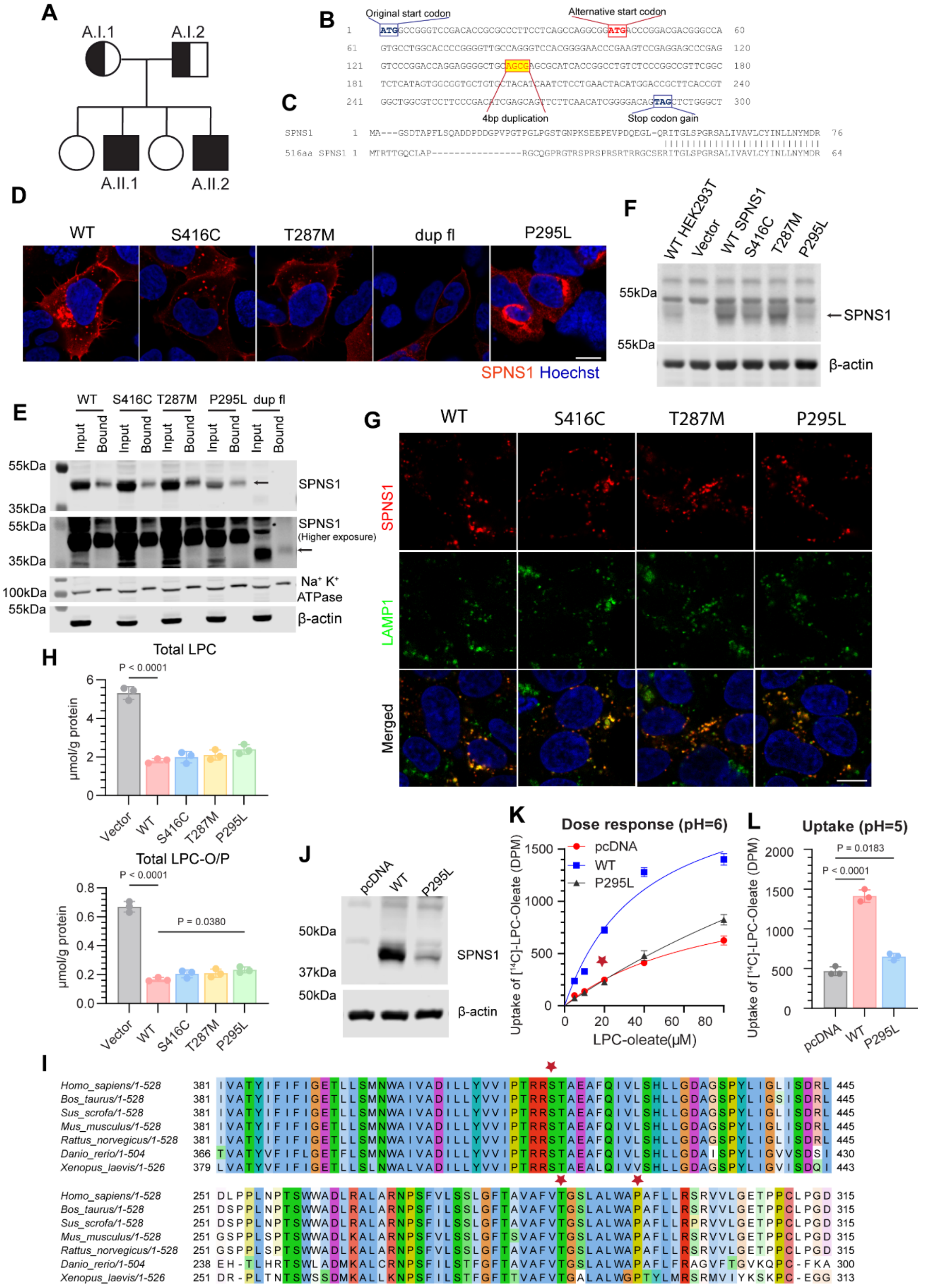

Supplemental Figure 1. Characterization of SPNS1 mutations. (**A**) Pedigree of Family A affected by *SPNS1* mutations. The father (square) is the carrier of c.(1247C>G), p[Ser416Cys] and the mother (circle) is the carrier of c.(143_146dupAGCG), p[Ile50Alafs*48]. (**B**) The cDNA sequence of the c.143_146dupAGCG variant. The location of the start codon, the duplication mutation, the stop codon gain, and location of the alternative start codon are indicated. (**C**) The N-terminal amino acid sequences of wild type SPNS1 and translation product from the alternative start codon (516aa SPNS1). The sequence after amino acid 64 of the 516aa SPNS1 is not shown as it is identical to WT SPNS1. (**D**) Immunofluorescence of WT SPNS1, T287M, S416C, c.143_146dupAGCG and P295L expressed in HEK293 cell. Scale bar =10 µm. (**E**) Representative image of three independent experiments for immunoblotting of cell surface levels of SPNS1 in HEK293 cell overexpression indicated SPNS1 constructs by capturing cell surface proteins through biotinylation. The bound fraction is the biotinylated cell surface proteins purified by streptavidin pulldown. The presence of Na^+K^+ATPase and absence of β-actin in the "bound" fraction indicated successful enrichment for cell surface proteins. A high exposure of the blot is shown because of low expression of the dup fl protein. (**F**) Immunoblotting of SPNS1 in rescued HEK293T cell lines. Arrow indicates location of SPNS1 band. (**G**) Immunofluorescence staining of SPNS1 (red) and LAMP1 (green) in rescued HEK293T cell lines. Scale bar: 10µm. (**H**) Lipidomics analysis on total cellular LPC and ether LPCs (LPC-O/P) level in rescued cell lines. (**I**) Multiple sequence alignment of seven *SPNS1* variants using MAFFT(1) and was visualized and coloured using Jalview ClustalX colour scheme(2). Stars denote the disease-causing human mutations discussed in this paper. (**J**) Immunoblotting of wild type SPNS1 (WT), P295L and vector control (pcDNA) expressed in HEK293 cell. (**K**) Concentration dependent transport of [^{14}C]-LPC-oleate by HEK293 cells overexpressing P295L mutant as compared to overexpressing WT *SPNS1* and vector control (pcDNA) over 30 minutes at pH=6 extracellular buffer. (**L**) Transport of [^{14}C]-LPC-oleate by HEK293 cells by pcDNA, WT SPNS1, and P295L at pH=5 extracellular buffer. n=3 replicates. Data are represented as mean±S.D. Statistical tests were one-way ANOVA with Dunnett's test when compared to WT for (**H**), and when compared to pcDNA for (**L**).

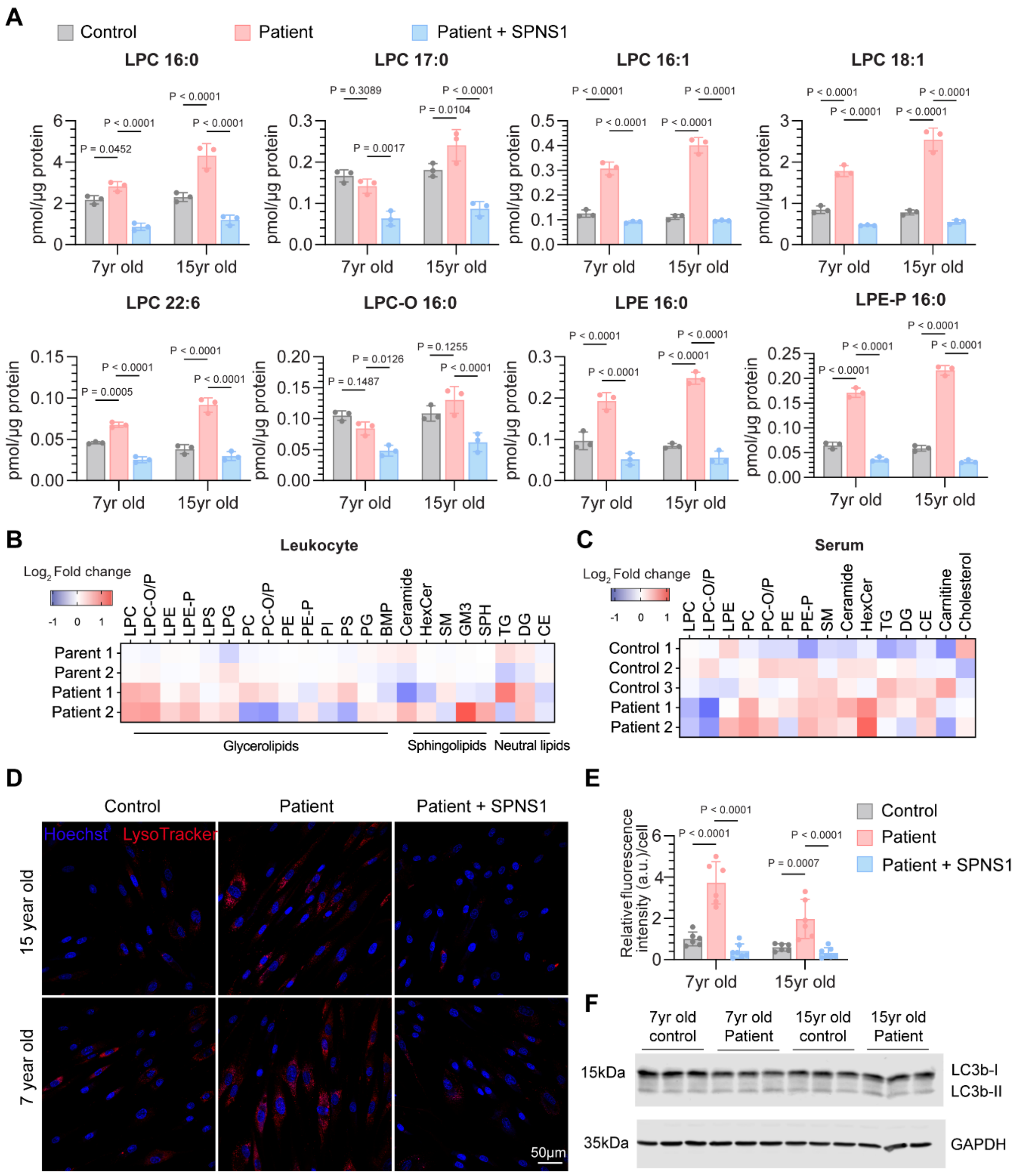

Supplemental Figure 2. Characterization of *SPNS1* patients' fibroblasts. (**A**) Concentrations of lysophospholipids in age-matched control fibroblasts transduced with vector control lentivirus (Control), patient fibroblasts transduced with vector control lentivirus (Patient), and patient fibroblasts transduced with WT *SPNS1* lentivirus (Patient+SPNS1) for five days. (**B**) Log_2 fold change of mole abundance of each lipid species among their respective lipid classes (glycerolipids, sphingolipids and neutral lipids) in leukocytes isolated from peripheral blood of patients as compared to their parents. (**C**) Log_2 fold change of concentration of each lipid in patients' serum samples as compared to control serum samples. (**D**) LysoTracker (red) staining of fibroblasts described in (**A**) 12 days after transduction. Blue: Hoechst. Scale bar: 50 µm. (**E**) Quantification of average LysoTracker staining intensity per cell in each field in (**D**). Each data point represents one field. At least six different fields were scored. (**F**) Immunoblotting of LC3b-I and LC3b-II from patients and control fibroblasts. n=3. Data are represented as mean± S.D. Statistical tests for (**A**) and (**E**) were two-way ANOVA with Dunnett's test.

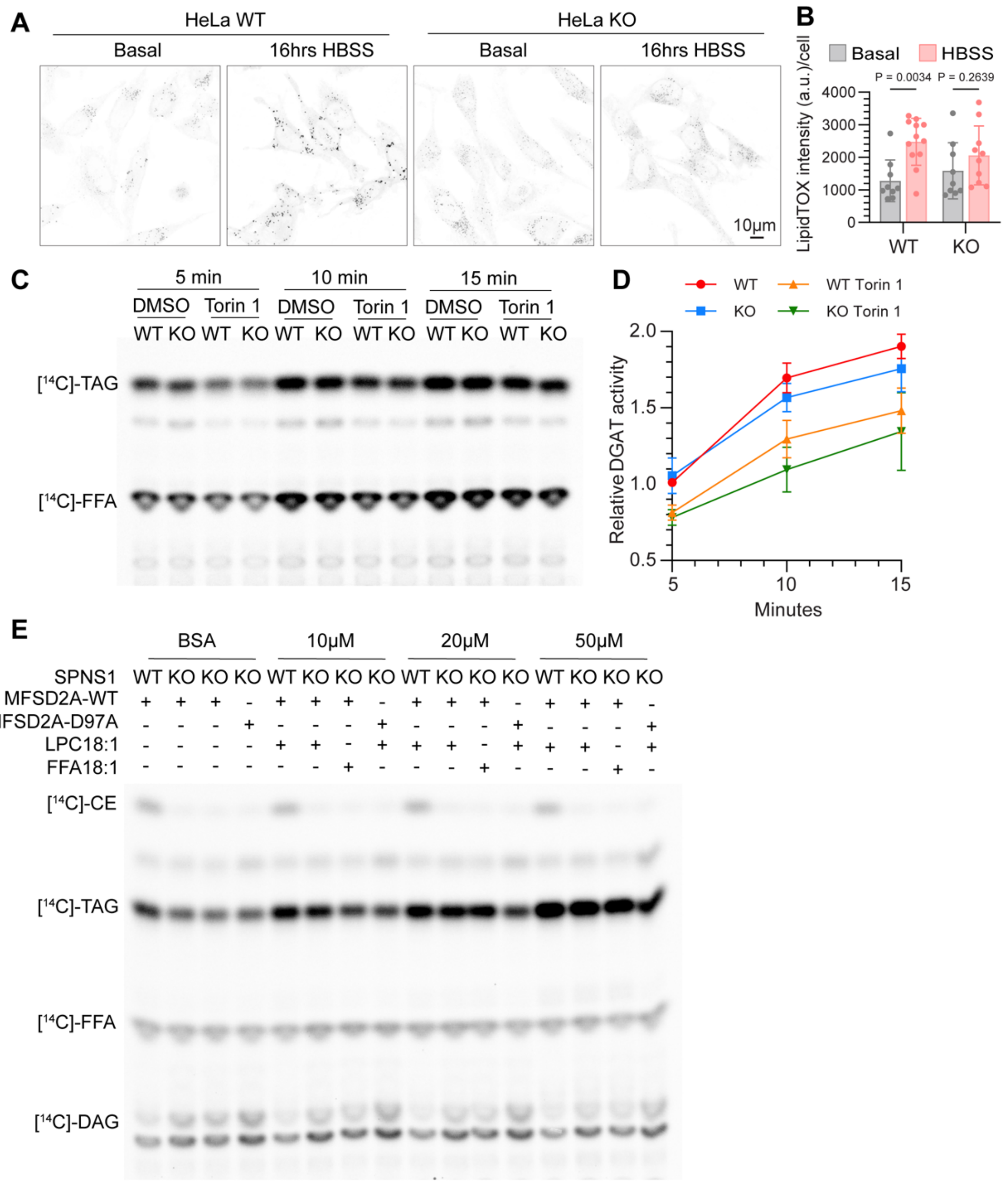

Supplemental Figure 3: Defective neutral lipid synthesis in *SPNS1* KO cells. (**A**) LipidTOX™ (stains lipid droplets) staining of WT and *SPNS1* KO HeLa cells cultured in full medium (basal) and after 16 hrs of HBSS treatment. Images were maximum projection of z-stack images. Scale bar: 10 μm. (**B**) Quantification of LipidTOX™ intensity per cell in each field in (**A**). Each data point represents one field and at least nine different fields were scored. (**C**) DGAT activity assay using membrane fractions isolated from HEK293T WT and *SPNS1* KO cells. Each reaction contains 18.5 μM [^{14}C]-oleoyl-CoA (FFA), 400 μM dioleoyl glycerol (DOG) and 50 μg of membrane fraction. Lipids were separated by TLC analysis. (**D**) Quantification of the [^{14}C]-TAG band intensity from (**C**), n=3 replicates. (**E**) Radioisotope tracing study as described in Figure 2G, except here three different concentrations of FFA-18:1 and LPC-18:1 were used. Data represents mean ± S.D. Statistical tests were two-way ANOVA with Šídák's test.

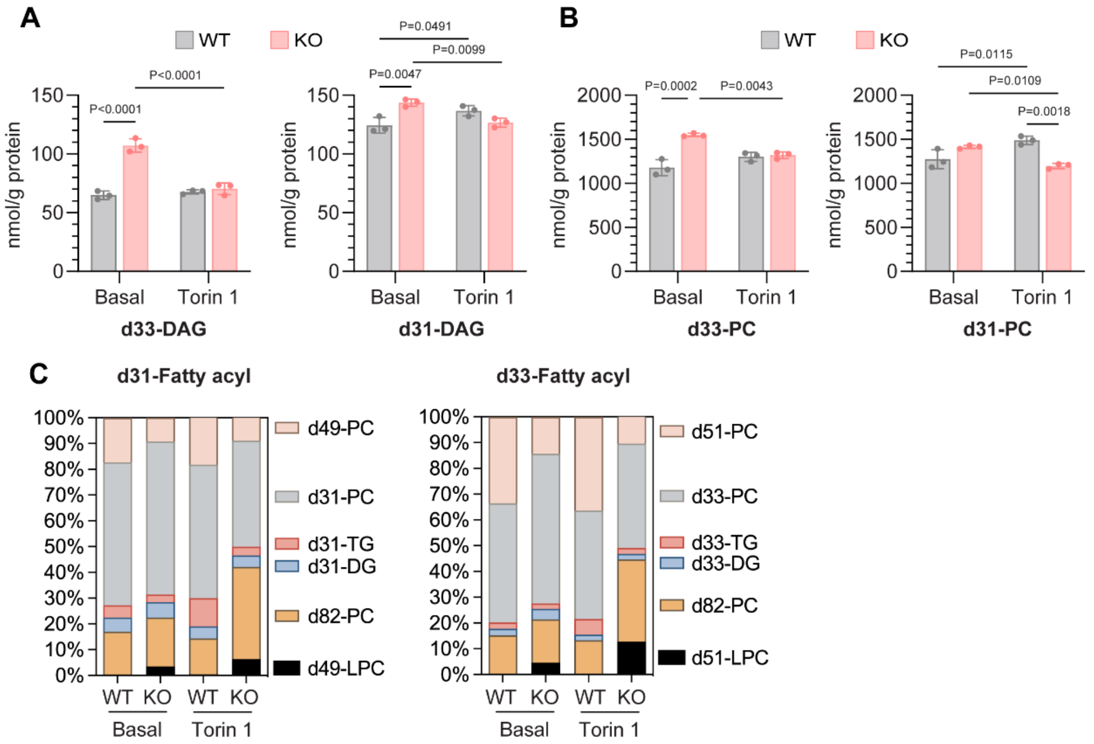

Supplemental Figure 4. Stable isotope tracing of lysosomal delivered d82-POPC. (**A**) Concentrations of all diacylglycerol (DAG) containing d31-FA-16:0 (d31-DAG) or d33-FA-18:1 (d33-DAG). (**B**) Concentrations of all PC species containing d31-FA16:0 (d31-PC) or d33-FA18:1 (d33-PC). (**C**) Distribution of d31-fatty acyl 16:0 and d33-fatty acyl 18:1 moiety among the indicated lipid classes. Percentage is calculated as the concentration of indicated lipid class over total concentration of all d31-FA, or d33-FA containing species within each sample. n = 3 replicates. Data are represented as mean ± S.D. Statistical tests were two-way ANOVA with Tukey's test.

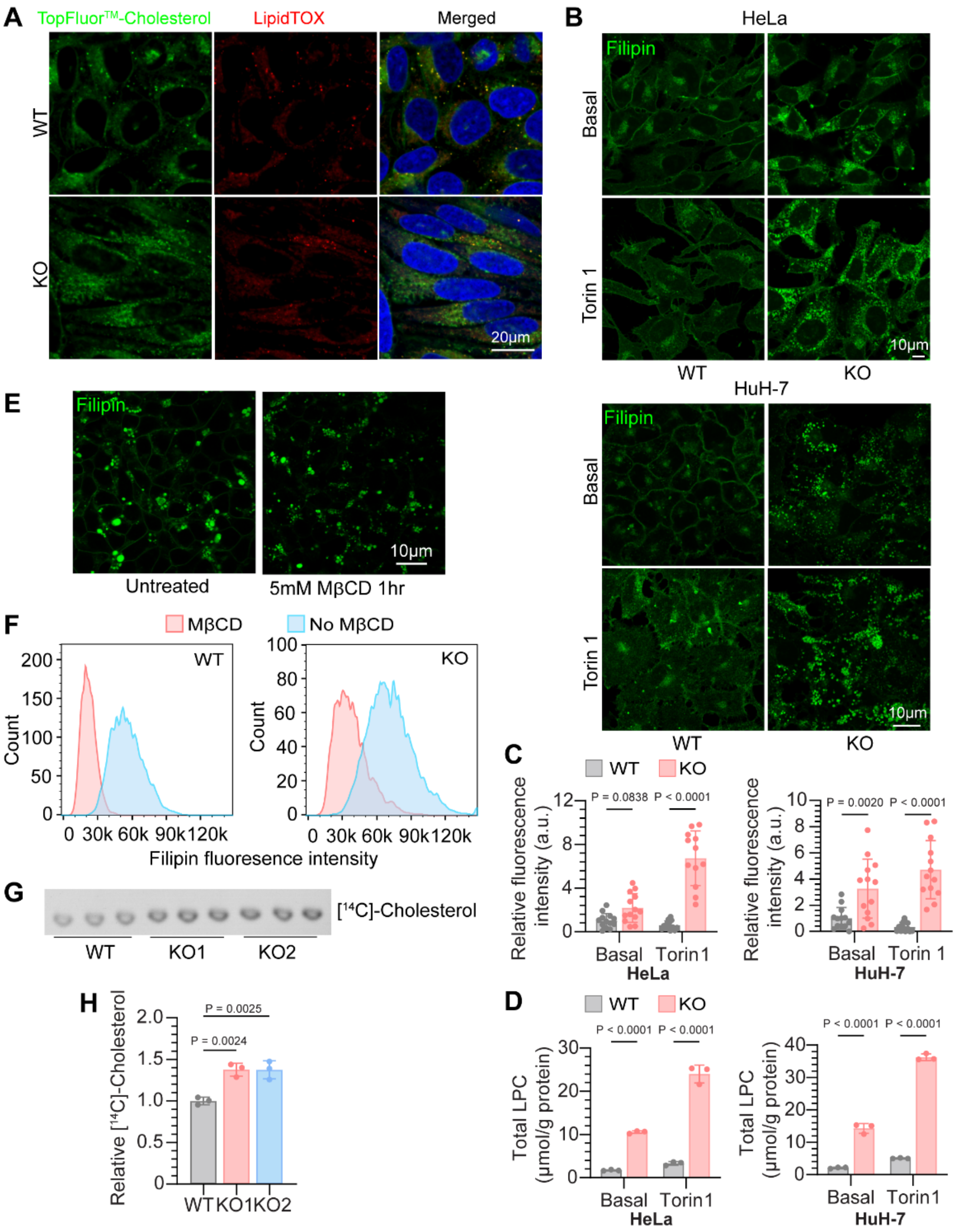

Supplemental Figure 5. SPNS1 deficient cells accumulate lysosomal cholesterol. (**A**) *SPNS1* KO and WT HeLa cells were labelled with TopFluor™-Cholesterol (Green) for 24 hrs followed by 16 hrs incubation in fresh medium. Fixed cells were stained with LipidTOX™ (red) for LDs. Blue: Hoechst. Scale bar: 20µm. (**B**) Filipin staining of WT and *SPNS1* KO HeLa cells or HuH-7 cells grown in full media (basal) or 16 hrs after 250nM Torin1 treatment. Scale bar: 10µm (**C**) Quantification of filipin staining intensity in each field of (**B**). At least 11 fields were scored. (**D**) Level of total LPCs in WT and *SPNS1* KO cells for treatment described in (**B**). (**E**) Representative images of filipin staining of HEK293T *SPNS1* KO cells with or without 1hr treatment of 5mM MβCD to deplete plasma membrane cholesterol. Scale bar: 10µm. (**F**) Flow cytometry profile of filipin staining of HEK293T *SPNS1* KO and WT cells with or without 1 hr treatment of 5mM MβCD. Filipin staining of WT and *SPNS1* KO HeLa cells. (**G**) [^{14}C]-cholesterol level in lysosomes isolated from HEK293T *SPNS1* KO and WT cells. Cells were pulsed labelled with [^{14}C]-cholesterol for 28 hrs and treated with 250 nM Torin1 in the last 12 hrs. Lipids were isolated from purified lysosomes and separated by TLC analysis. (**H**) Quantification of relative [^{14}C]-cholesterol band intensity in (**G**) after normalizing to protein amount for each lysosome sample. Data are represented mean ± S.D. Statistical tests were two-way ANOVA with Šídák's test for (**C**) and (**D**), and one-way ANOVA with Dunnett's test for (**H**).

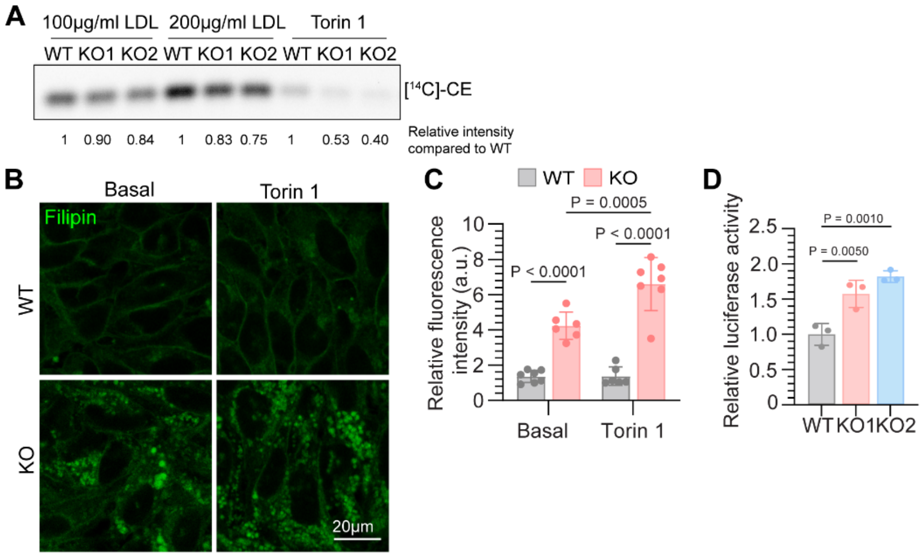

Supplemental Figure 6. SPNS1 deficient cells accumulate lysosomal cholesterol. (**A**) Quantification of CE synthesis in HEK293T WT and *SPNS1* KO cells using metabolic labelling with [^{14}C]-oleate. Cells were grown in delipidated medium for 20 hrs and changed to delipidated medium with 100 µg/ml or 200 µg/ml human low density lipoprotein (LDL) with 50 µM mevastatin and 50 µM mevalonate or 10% FBS containing medium with 250 nM of Torin 1 for another 24 hrs. In the last 2 hrs of treatment, cells were labelled with 50 µM of oleate containing a trace amount of [^{14}C]-oleate. Lipids were separated by TLC and [^{14}C]-CE band intensities were quantified and represented as relative to WT levels. (**B**) Filipin staining of HeLa WT or *SPNS1* KO cells after loading cells with LDL. Cell were delipidated for 24 hrs followed by 24 hrs treatment with 50 µg/ml LDL. For Torin 1 treatment, 250 nM of Torin 1 was added in the last 12 hrs of treatment. Basal indicates cells not treated with Torin 1. Scale bar: 20µm. (**C**) Quantification of filipin staining intensity from panel (**B**). At least 6 fields were scored. (**D**) Luciferase activity of a sterol response element construct in HEK293T *SPNS1* KO and WT cells after 24 hrs of delipidation. n=3 replicates. Data are represented as mean ± S.D. Statistical tests were two-way ANOVA with Šídák's test for (**C**) and one-way ANOVA with Dunnett's test for (**D**).

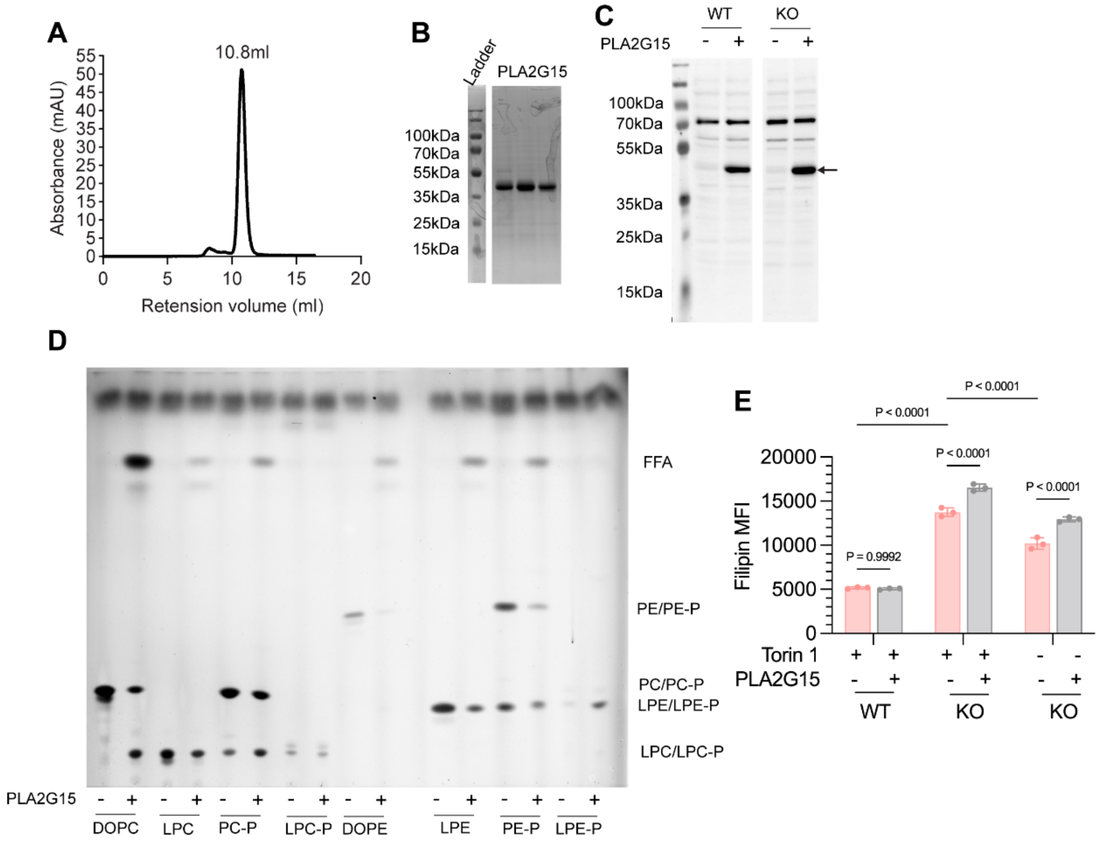

Supplemental Figure 7: Purification of PLA2G15 and its effect on cellular cholesterol levels. (**A**) High-performance liquid chromatography elution profile of PLA2G15-6His. (**B**) Representative SDS-PAGE of purified PLA2G15-6His. (**C**) Demonstration that HEK293T cells take up recombinant PLA2G15-6His. HEK293T cells were incubated with 3 µg/mL purified PLA2G15-6His for 48 hrs then immunoblotted for PLA2G15-6His. Arrow indicates the location of His-tagged protein. (**D**) TLC analysis was used to measure lipase activity of PLA2G15-6His at pH = 4.5 using the following lipid substrates: 1,2-Dioleoyl-sn-glycero-3-phosphocholine (DOPC), C18(Plasm)-18:1 PC (PC-P), 18:1 LPC, C18(Plasm) LPC (LPC-P), 1,2-Dioleoyl-sn-glycero-3-phosphoethanolamine (DOPE), C18(Plasm)-18:1 PE (PE-P), 18:1 LPE, C18(Plasm) LPE (LPE-P). The identity of the relevant lipids and fatty acid product (FAA) are indicated. (**E**) Flow cytometry measurement of the geometric mean of filipin fluorescence intensity in WT and HEK293T *SPNS1* KO cells with or without 48 hrs of PLA2G15-6His supplementation, followed by 8 hrs of with or without Torin1 treatment; n=3 replicates. Data are represented as mean ± S.D. Statistical tests were two-way ANOVA with Tukey's test.

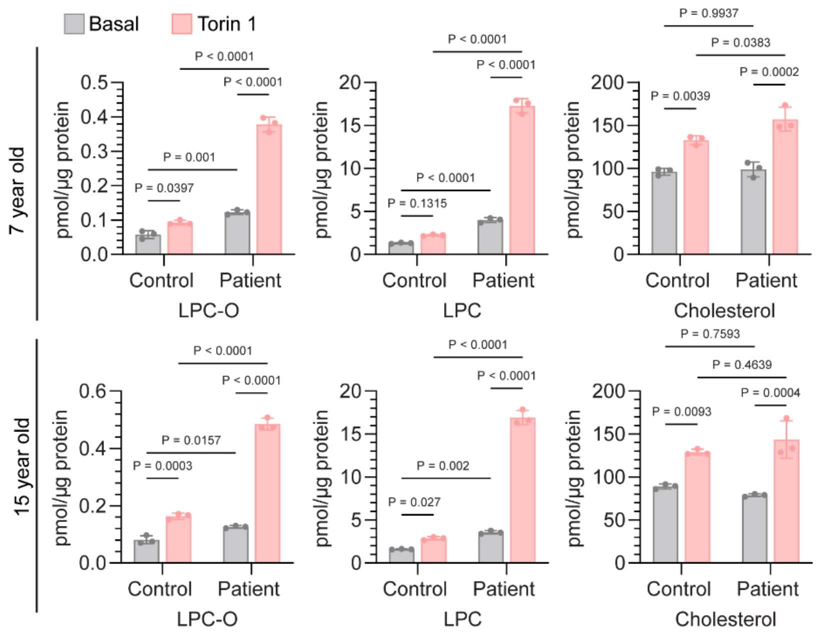

Supplemental Figure 8: Lipidomic analysis of patient fibroblasts under mTOR inhibition. Patients and their age-match control fibroblasts were cultured either in basal condition or with 16 hrs treatment with 250 nM Torin 1. Before harvesting, cells were treated with 5 mM of MβCD for 1 hr to deplete plasma membrane cholesterol. n=3 replicates. Data are represented as mean ± S.D. Statistical tests were two-way ANOVA with Šídák's test.

Supplemental methods

Cell culture

HEK293T and HeLa cells were obtained from American Type Culture Collection (ATCC) and routinely grown in high glucose DMEM (HyClone) supplemented with 10% heat inactivated FBS (Gibco) and penicillin/streptomycin (Gibco) at 37 °C with 5% CO_2. HuH-7 cells (JCRB0403) were obtained from the RIKEN BRC Cell Bank and maintained in the same medium except low glucose DMEM was used. Patient derived dermal fibroblasts were cultured in MEM (Gibco) supplemented with 10% FBS, 1% GlutaMax, penicillin and streptomycin. FBS was delipidated as described (3). For experiments that involved delipidation condition, 5% delipidated FBS in DMEM was used. HEK293S GnT1-/- cells were maintained in FreeStyle 293 medium (Gibco) supplemented with 2% FBS, penicillin/streptomycin and maintained in shaking incubator at 37 °C with 5% CO_2.

Genetic analysis

For family A: Exome sequencing (ES) was performed at Clinical Genomics, Stockholm, Sweden using Agilent SureSelect All Exon v4-7, Focused Exome or Twist Human Core Exome using Illumina HiSeq X Ten or Illumina Nova Seq platform. Nucleotide variants were called using Mutation Identification Pipeline (MIP) [https://github.com/Clinical-Genomics/MIP]. Genome sequencing (GS) was performed at Clinical Genomics, Stockholm, Sweden using the Illumina HiSeq X Ten, NovaSeq or NovaSeq X platform, using a 30× PCR-free paired-end WGS protocol. Nucleotide variants were called using Mutation Identification Pipeline (MIP).

Variants (SNVs and INDELs) were filtered for *de novo* variants, X-linked inheritance, and autosomal recessive inheritance (homozygous or compound heterozygous) in the family. Only sequence variants with an allele frequency below 1% in the general population, affecting exons or exon-intron boundaries were taken into consideration. In the case of autosomal recessive inheritance two variants in the same gene had to be present in order to be considered.

For family B: Exome sequencing was performed as previously described(4) in a diagnostic setting but the patient was also included in a study approved by the local ethics committee of the

Technical University Munich (5360/121S). Both parents gave written informed consent and the study was conducted in compliance with the declaration of Helsinki.

Generation of expression plasmids

Human codon optimized cDNAs for human *SPNS1* was synthesized by GenScript® and 4bp duplication mutation in *SPNS1* cDNA was synthesized by IDT. All of the cDNAs were cloned into pcDNA3.1 plasmid respectively. *SPNS1* variants were generated by the QuickChange site-directed mutagenesis method (Agilent) (5). Viral mediated rescue plasmids for patient fibroblasts were generated by cloning *SPNS1* sequences followed by an IRES sequence into FUGW plasmid (Addgene, 14883). For rescue plasmid used for HEK293T *SPNS1* KO cells, WT and mutant *SPNS1* constructs were driven by thymidine kinase promoter and inserted into AAVS1 vector with hygromycin selectable marker (AAVS1-pTK-SPNS1-Hygro).

Cell surface transport assay

HEK293 cells grown in 24-well plates were transfected in with 0.5 µg/well of plasmid encoding indicated constructs (in triplicate) for 20 hrs using Lipofectamine™ 2000 (Thermo Fisher). Cells were washed once with 0.5 mL reaction buffer (25 mM sodium citrate, 150 mM NaCl, 5 mM glucose, 1 mM $MgCl_2$ for pH=5 and 25 mM Na MES, 150 mM NaCl, 5 mM glucose, 1 mM $MgCl_2$ for pH=6) followed by incubation with indicated concentration of radiolabeled 1-oleoyl 2-lysophosphocholine ([^{14}C]-LPC oleate, specific activity: 55 mCi/mmol, from American Radiolabeled Chemicals) in 0.25 mL reaction buffer for 30 min at 37 °C with 5% CO_2. Cells were washed twice with 0.3 mL of 0.5% (w/v) fatty-acid free BSA (Sigma) in reaction buffer, then extracted twice with 0.5 mL of 1% Triton X-100 in Tris-buffered saline. Cell lysates were transferred to 4 mL of EcoLite(+)™ scintillation fluid (Avantor) for scintillation counting using a Tri-Carb® 2910 TR Liquid Scintillation Analyzer (PerkinElmer). [^{14}C]-LPC oleate was dried by blowing with argon stream and resuspended in 20 mM of the corresponding unlabeled lipid (Avanti) in 12% (w/v) fatty-acid free BSA as previously described (6).

Sulfo-NHS- biotin cell surface labeling

HEK293 cells grown in 6-well plates were transfected with 1 µg/well of respective constructs for 20 hrs, washed twice with cold PBS+ (10mM $MgCl_2$, 0.09mM $CaCl_2$, pH7.4), and incubated with 1mL of 0.75 mg/mL EZ-Link™ Sulfo-NHS-biotin (Thermo Fisher, 21217) in PBS+ on ice with gentle mixing for 2 hrs. the labeling solution was removed and cells washed twice for 10min each with ice cold 100mM glycine in PBS+. Cells were lysed in RIPA (Thermo Fisher) supplemented with cOmplete protease inhibitor. Lysates were cleared by centrifuging at 16000 x g for 15min. 35 µg of total cell lysate was used as input fraction. 350 µg of lysate was bound to 25 µL (packed volume) of Strep-Tactin® Superflow® resin (IBA, 2-1206-025) overnight. Beads were washed twice with RIPA buffer and twice with 10x diluted PBS. Bound protein on beads (i.e. biotinylated) were incubated with 25 µL of 2x SDS loading buffer to generate the bound fraction.

Generation of cell lines

SPNS1 KO and *SPNS1 PLA2G15* DKO cell lines were generated with CRISPR-Cas9 approach. sgRNA targeting sequence of *SPNS1* is 5'-CTACATGGACCGCTTCACCG-3' and *PLA2G15* is 5'- GCTGCTGCTAATGCTGCTCG-3', respectively. sgRNA oligos were annealed, cloned into pX330-mCherry plasmid (Addgene, 98750) and transfected into HEK293T, HeLa expressing MFSD2A stable cell lines and HuH-7 cells. mCherry expressing cells were sorted to a single clone and gene KO was validated by Sanger sequencing. For HEK293T and HeLa cells, single clones were used for experimentation. For the HuH-7 cells, a mixed clone cell line was used in experiments. *SPNS1 PLA2G15* DKO were generated from the *SPNS1* KO line.

Rescue experiment for patient fibroblasts was performed by lentiviral transduction. Lentivirus particles were produced by transfecting HEK293T cells in with FUGW-SPNS1-IRES or FUGW-IRES, pMDLg/pRRE, pRSV-Rev (Addgene, 12253), and pCMV-VSV-G (Addgene, 8454), with Lipofectamine 2000. Viral medium was collected at 48 hrs and 72 hrs post transfection and passed through a 0.45 µm filter. Polybrene (Sigma) was added at 8 µg/mL, and 1 mL of virus medium was used to infect patient fibroblasts seeded in 6-well plates. Cells were harvested 5- or 12-days post infection.

HEK293T WT, *SPNS1* KO and HeLa cells with inducible *MFSD2A* expression was generated by lentivirus transduction. Lentivirus particles were produced by transfecting HEK293T cells with TetOn-MFSD2A-GFP or TetOn-MFSD2A (D97A)-GFP, pMDLG/PRRE (Addgene, 12251), pRSV-Rev (Addgene, 12253), and pCMV-VSV-G (Addgene, 8454) plasmids with Lipofectamine 2000. Media containing lentiviral particles were collected at 48 hrs and filtered with a 0.45 µm membrane filter. Polybrene was added to a final concentration of 8 µg/mL. 0.5µg/mL or 2µg/mL of puromycin was added to medium 48 hrs after transduction of HEK293T or HeLa cell lines for selection, respectively. HEK293T rescue cell lines were generated by co-transfecting HEK293T *SPNS1* KO cells with both AAVS1-pTK-SPNS1-Hygro plasmid and pCas9-sgAAVS1-1(Addgene, 129726). Two days post transfection, cells were selected using 200 µg/mL of Hygromycin B (Nacalai) for 14 days.

Isolation of lysosomes using superparamagnetic iron oxide nanoparticles (SPIONs)

Isolation of lysosomes from cultured cells were carried according to (7). Briefly, cells grown in 10 cm dishes were pulsed with medium containing 10% dexoMAG 40 SPIONs (Liquids Research Limited) for 24 hrs, then chased in complete medium for another 24 hrs. Cells were collected in 2mL of homogenization buffer (HB) (250 mM sucrose, 10 mM HEPES, 1 mM $CaCl_2$, 1 mM $MgCl_2$, 1.5 mM Mg Acetate, cOmplete protease inhibitor, pH=7.4) and dounced 25 times in a glass homogenizer. The homogenate was centrifuged at 200x g for 10 min, supernatant collected, and the pellet was dounced again in 1 mL of homogenization buffer for 25 times, centrifuged again at 200x g for 10 min. The combined post-nuclear supernatant was passed through a Miltenyi LS Separation column on MidiMACS Magnetic Separator to allow SPIONs containing lysosomes to bind. The column was washed with 1 mL of HB containing 10 µg/mL DNase and another 3 mL of HB. Lysosomes were eluted with 500 µL of HB twice.

Radioisotope labeling of cells

For pulse labeling of cells with [^{14}C]-oleic acid (American Radiolabeled Chemicals, Specific activity: 55 mCi/mmol), cells in 12-well plates were first grown in complete medium and treated

with 250 nM Torin 1 for the times indicated. In the last 4 hrs of treatment, medium containing 0.25 µCi/mL of [^{14}C]-oleic acid was added. For experiments with inducible MFSD2A, cells were pretreated with 1 µg/ml doxycycline for 8 hrs. Medium was then replaced by medium containing 1 µg/mL doxycycline and 250 nM Torin 1 for another 16 hrs. Medium containing 0.25 µCi/mL of [^{14}C]-oleic acid and indicated concentration of FFA 18:1 and LPC-18:1 conjugated to 12% (w/v) fatty acid free BSA or equivalent volume of fatty acid free BSA for 4 hrs. For experiments with cholesterol supplementation, after Torin 1 treatment, cells were washed twice with PBS and treated with delipidated medium containing 0.25 µCi/mL of [^{14}C]-oleic acid and 35.7 µM cholesterol complexed with MβCD (1:10 molar ratio) with or without 20 µM of FFA 18:1 was added for 4 hrs. For LDL loading experiment, cells were seeded on day 0 and changed to medium containing delipidated FBS on day 1. On day 2, medium was change to delipidated medium containing 50 µM mevastatin and 50 µM Li mevalonate with either 100 µg/mL or 200 µg/mL of LDL, or FBS containing medium with 250 nM Torin 1 for another 24 hrs. On day 3, two hrs before harvesting, a final concentration of 0.25 µCi/mL of [^{14}C]-oleic acid and 50 µM of FFA 18:1 was added to cells. Cells were washed twice with 0.5% (w/v) fatty-acid free BSA in PBS to remove excess [^{14}C]-oleic acid prior to lipid extraction. Cells were dried on the plate and extracted twice with 500 µL of hexane: isopropanol 3:2 (v/v) for 30 minutes. The two extracts for each sample were combined and dried under a nitrogen stream. The remaining cells were lysed with 250 µL cell lysis buffer (0.1% SDS, 0.1M NaOH) and protein concentration was measured with BCA assay (Thermo).

For [^{14}C]-cholesterol labeling of cells, cells were labelled with 0.05 µCi/mL of [^{14}C]-cholesterol (American Radiolabeled Chemicals, Specific activity: 55 mCi/mmol) during the 28 hrs chase period after pulsing with SPION for 24 hrs and in the last 12hrs, Torin 1 was added to a final concentration of 250 nM. One mL of isolated lysosome fraction was extracted with four mL of Chloroform: methanol 2:1 (v/v), vortex for 30 minutes and centrifuge at 2000 xg for 5 mins. The lower organic phase was dried for subsequent TLC analysis.

DGAT activity assay

HEK293T WT and *SPNS1* KO cells were grown in 10 cm dish and treated with or without 250 nM Torin 1 for 16 hrs. Cell pellets were harvested and resuspended in 1 mL of sucrose buffer (50 mM Tris-HCl, pH 7.6, 250 mM sucrose). Cells were lysed by passing through 27g needle for 35 times. After removing nuclei and un-lysed cells, the supernatant was ultracentrifuged at 100,000 xg for 30 mins. The pellet was resuspend in sucrose buffer to achieve a protein concentration of 1 mg/mL. Each reaction contained 20 μL of 1 M Tris-HCl (pH 7.6), 4 μL of 1 M $MgCl_2$, 10 μL of 4 mM dioleoyl glycerol (DOG),10 μL of 12.5 mg/mL BSA, 10 μL of 363.6 μM [^{14}C]-oleoyl-CoA, 50 μL of membrane fraction and 96 μL of water per reaction. The reaction mixture was incubated at 37°C for indicated times and terminated by adding 800 μL chloroform: methanol (2:1), vortexed for 30 mins and centrifuged 2000 xg for 5 mins. The lower organic phase was extracted and dried for TLC analysis.

Thin Layer chromatography

Dried lipid films were reconstituted with chloroform: methanol 3:2 (v/v) and spotted on Silica glass plate (Merck, 1.05721.0001). The mobile phase for TLC was chloroform: methanol: water 65:25:4 (v/v) for phospholipids and hexane: diethyl ether: acetic acid 80:20:1 (v/v) for neutral lipids. For radioisotope labelling experiments, TLC plates were exposed overnight to Phosphorimager screens and scanned using a Typhoon FL A9500 and lipid band intensities were quantified using ImageLab and normalized to protein concentration or total lane intensity as indicated. For enzyme assay, silica plate was charred with 5% $CuSO_4$ in 15% Phosphoric acid followed by 10mins incubation in 180 °C oven.

Cell fractionation for SREBP2 processing

Cell fractionation was performed as described(3). In brief, HEK293T cells grown in 10cm dish were treated with 25 μg/mL ALLN 2hrs before harvesting. Washed cell pellets were resuspended in buffer A (250 mM sucrose, 10 mM HEPES-KOH at pH 7.6, 10 mm KCl, 1.5 mM $MgCl_2$, 1 mM sodium EDTA, 1 mM sodium EGTA, 2.8 μg/mL aprotinin, 10 μg/mL leupeptin, 25 μg/mL ALLN, 5 μg/mL pepstatin A, and 0.5 mM Pefabloc). Cells were homogenized by passing through a 23g

needle 25 times and centrifuged at 1000 xg for 5 mins. The 1000 xg pellet was resuspended in 0.1 mL of buffer B (20 mM HEPES-KOH at pH 7.6, 420mM NaCl, 2.5% (v/v) glycerol, 1.5 mM MgCl2, 1 mM sodium EDTA, 1 mM sodium EGTA, and the above mixture of protease inhibitors), rotated for 1hr at 4 °C and centrifuged at 100,000 xg for 15 mins and resulting supernatant was designated as nuclear fraction. The 1000 xg supernatant was centrifuged at 100,000 xg for 15 mins and the pellet was resuspended in SDS lysis buffer (10 mM Tris-HCl at pH 6.8, 100 mM NaCl, 1% (w/v) SDS, 1 mM sodium EDTA, 1 mM sodium EGTA, and the above mixture of protease inhibitors) and designated as the membrane fraction.

Luciferase assay

HEK293T WT or KO cells grown in 12-well plates were transfected with 0.5 µg of pSynSRE-T-Luc (addgene, 60444) and 5 ng of pRL-renilla luciferase (Promega) using Lipofectamine 2000. After 6 hrs, medium was replaced with DMEM containing 5% delipidated FBS and incubated for 16hrs. Cells were then treated with 250 nM Torin 1 either with delipidated medium or FBS containing medium for 8hrs. Cells were harvested and luminescence was measured with the Dual-Luciferase Reporter Assay System (Promega) according to the manufacturer's instruction. Luminescence was quantified using a Tecan Infinite M200 Microplate Reader. Relative luciferase activity was expressed as a ratio of firefly luminescence to renilla luminescence.

ApoE-d82 POPC-36:2 nanodisc generation and cell treatment

1 mg of d82-POPC 34:1 (Avanti, 860320) was conjugated to 500 µg of apoE3 (Sigma, 900010) as previously described(8). Cells were grown in delipidated medium for 24 hrs before adding 15 µg/mL apoE-d82-POPC-34:1 nanodiscs with or without 250nM Torin 1 for another 16 hrs. Lipid and protein were extracted as described in the previous section.

Immunoblotting

Whole cells were lysed with RIPA buffer (Thermo Fisher) supplemented with cOmplete protease inhibitor cocktail. Protein concentration was determined by BCA assay. Lysates were denatured

in SDS-PAGE loading buffer containing 2.5% β-mercaptoethanol (BME). Protein was separated by SDS-PAGE and transferred to 0.45 µm or 0.22 µm nitrocellulose membranes. Membranes were blocked with 5% non-fat milk in TBS-T (50 mM Tris, 150 mM NaCl, 0.1% Tween-20) for 1 hr at room temperature and blotted with primary antibodies in 5% BSA in TBS-T overnight. Antibodies used were anti-LC3B (Cell signaling, 2775S), anti-SREBP2 (Merck Millipore, MABS1988), anti-Lamin B1 (Santa Cruz, sc-374015), anti-6x-His (Bethyl labotoaries A190-114A), anti-beta actin (Sigma, A2228), anti-GAPDH (6C5) (Santa Cruz, sc32233), anti-SPNS1 (HPA041995) and anti-Sodium Potassium ATPase Alpha 1 Antibody (464.6) (Novus Biologics, NB300-146). Rabbit polyclonal antibodies were raised against human SPNS1 C-terminal amino sequence GRSTRVPVASVLI (in house). All primary antibodies were used at 1:1000 dilution. Secondary antibodies used are RDye® 800CW Donkey anti-Rabbit IgG (LiCOR, 926-32213) and IRDye® 680LT Donkey anti-Mouse IgG (LiCOR, 926-68022). Secondary antibody (1:10000 dilution) in 5% BSA in TBS-T was used for detection using an ODYSSEY infrared imaging system (LI-COR).

Immunofluorescence microscopy

Cells were fixed with 4% PFA for 10 mins and permeabilized and blocked with 0.1% Saponin and 5% normal goat serum (NGS) in PBS for 30 mins at room temperature. Cells were then incubated with primary antibody in 0.1% Saponin and 2% NGS overnight at 4 °C. After washing, cells were incubated with AlexaFluor secondary antibody (1:400 dilution) for 1 hr. Primary antibody dilutions were 1:800 for LAMP1 (DSHB, H4A3) and 1: 400 for SPNS1. Nuclei were counter-stained with Hoechst 33342 (1:1000) for 5 mins. Images were obtained using a LSM710 Confocal Microscope (Carl Zeiss).

LysoTracker Staining and quantification

Cells were grown in 8-well glass chamber slides, stained with 200 nM LysoTracker™ Red DND-99 (Thermo Fisher, L7528) in medium for 30 mins. Hoechst 33342 (1:1000) (Thermo Fisher) was added to medium during the final 10 mins of incubation. Cells were washed once with PBS and

fixed with 4% paraformaldehyde for 15 mins. Cells were imaged using LSM710 Confocal Microscope (Carl Zeiss). LysoTracker fluorescence intensity after thresholding to remove background was calculated using ImageJ Fuji either for individual cell or for entire image field. When fluorescence intensity for the entire field is used, it is normalized to the number of nuclei in the field.

Staining of lipid droplets and quantification

LDs in HEK293T Cells were incubated with 5 μM of BODIPY™ 493/503 for 30 mins in culture medium. Excess dye was washed away, and cells were incubated further with 50 nM LysoTracker and Hoechst for 15min before imaging as noted above. Number of lipid droplet (LD) in the entire image field was calculated using Analyze Particle in ImageJ after thresholding to remove background fluorescence and normalized to the number of nuclei in the field.

LD in HeLa cells were stained HCS LipidTOX Neutral Lipid Stain (Thermo Scientific, H34476) at 1:500 dilution in PBS for 2 hrs after fixation. Nuclei were counterstained with Hoechst. Z-stack images at 1 μm interval were captured. Images are max projections of z-stack and total fluorescence of the image field was calculated and normalized to number of nuclei in the field.

TopFluor™ Cholesterol labeling of cells

For live cell imaging, TopFluor™ Cholesterol (Avanti, 810255) stock solution (1 mM) was prepared by dissolving dried TopFluor™ Cholesterol in 185 mM methyl-beta cyclodextrin (MβCD) (Sigma, 332615). Cells were washed and labelled with 1 μM TopFluor™ Cholesterol in delipidated medium for 1 hr. Medium was then replaced by full medium containing 250 nM Torin 1 for 16 hrs. Cells were staining with 50 nM LysoTracker and Hoechst for 15 mins before imaging. For imaging of fixed cells, cells were labelled with 1 μM of TopFluor™ Cholesterol dissolved in DMSO in delipidated medium for 24 hrs, followed by treatment with or without 250 nM Torin 1 in full medium for 16 hrs before fixation and staining with LipidTOX.

Filipin staining of cells and image analysis

For imaging studies, cells were fixed with 4% paraformaldehyde for 15 mins and stained with 50 μg/mL filipin (Sigma, F9765) in PBS for 1 hr. For MβCD treatment, cells were washed with PBS and incubated with 5 mM MβCD in DMEM for 1 hr before fixation. For LDL loading experiment, HeLa cells were delipidated for 24 hrs before addition of 50 μg/mL of human LDL (STEMCELL technology, 02698) for another 24 hrs. 250nM of Torin 1 was added in the last 12 hrs of LDL loading for Torin 1 treated samples. Filipin fluorescence intensity after thresholding to remove background was calculated using ImageJ Fuji for entire image field and normalized to number of cells in the field when indicated.

Flow cytometry analysis

Cells from 6-well plates were harvested and washed cell pellet were incubated with 5 mM MβCD in DMEM on shaker in a 37 °C incubator with 5% CO_2 for 1 hr. Cells were then washed and fixed with 2% PFA for 30 mins at room temperature before staining with 50 μg/mL Filipin in FACS buffer (0.5% BSA in PBS) for 90 mins on a rotator at room temperature. Cells were washed twice with FACS buffer before flow cytometry using a BD FACS Celesta Cell Analyser. Single cells were gated and geometric mean fluorescence of filipin is quantified.

RT-qPCR

RNA from cells were extracted using RNeasy Mini Kit (74106, QIAGEN) according to the manufacturer's recommended protocol and reverse transcribed using iScriptTM Reverse Transcriptase Supermix (Bio-Rad). SensiFAST^{TM}SYBR® Hi-ROX Kit (Bioline) was used for qPCR and level of mRNA normalized to TATA-binding protein (TBP). Primers used are 5'-TTGTACCGCAGCTGCAAAAT-3' and 5'-TATATTCGGCGTTTCGGGCA-3' for human-*TBP*; 5'-GGGAACCTCGGCCTAATGAA-3' and 5'-CACCACGCTCATGAGTTTCCA-3' for human-*HMGCR*; 5'-CTCTTGGGATGGACGGTATGC-3' and 5'-GCTCCAACTCCACCTGTAGG-3' for *HMGCS*.

Expression and purification of PLA2G15 protein

HEK293S GnT1-/- cell were grown to 2-2.5x10^6/mL in Freestyle293 medium and transfected with 1 µg of pRK5-PLA2G15-6xHis WT (Addgene, 213603) per mL of cells using PEI at 1:4 DNA to PEI ratio. Transfection mixture was incubated with cells for 30 mins without shaking. 10 µM sodium butyrate was added 24 hrs after transfection and culture supernatant was harvested 72 hrs post-transfection. The filtered supernatant was concentrated to 1/3-1/4 of the original volume and bound to Ni-NTA resin (Qiagen) for 1 hour at 4 °C in the presence of 5 mM imidazole. Beads were then washed with 5 column volumes (CV) of high salt buffer (20 mM HEPES pH 7.4, 500 mM NaCl, 20 mM Imidazole, 0.05% Triton X-100), followed by 5 CV normal salt buffer (20 mM HEPES pH7.4, 150 mM NaCl, 10 mM Imidazole) and then eluted with 5 and 2.5 CV elution buffer (20 mM HEPES pH7.4, 150 mM NaCl, 300 mM Imidazole, 5 mM Beta-mercaptoethanol (BME)). The eluted protein was concentrated using an Amicon 10 kDa spin column and further purified by size exclusion chromatography using Superdex 75 10/200 in FPLC buffer (20 mM HEPES pH7.4, 150 mM NaCl, 5mM BME). Peak monodispersed fractions were collected and concentrated to 0.5-1 mg/mL and 5% glycerol were added before snap-freezing the protein in lipid nitrogen for storage.

Lipase supplementation

HEK293T cells were seeded at 5 x10^4 cells/mL in 12-well plate. 24 hrs later, cells were treated with 3 µg/mL of purified PLA2G15 for another 48 hrs before harvesting cells for subsequent analysis. Cells were incubated with 5 mM MβCD for 1 hour before harvesting for lipidomic analysis.

Phospholipase assay

Indicated substate lipids in chloroform stock were dried under nitrogen gas and hydrated with water to 2 mM for 30 mins with shaking. The lipid emulsion was sonicated for 10 mins. Each reaction (200 µL) contained 100 nM of PLA2G15 and 40 µM of lipid substrate in buffer containing 50 mM sodium acetate, 150 mM NaCl at pH=4.5 at 37°C with shaking for 1 hour. Reactions were stopped by adding 800 µL of chloroform: methanol 2:1 (v/v). After 30 mins, samples are

centrifuged for 5 mins at 2000 xg and the lower organic phase was extracted and dried for TLC analysis.

Sample preparation for lipidomics

For whole cells, dried plates of cells were extracted twice with 500 uL of hexane: isopropanol 3:2 (v/v) for 30 mins twice. The two extracts for each sample were combined and dried under a nitrogen stream. Dried cell extracts were resuspended with 200 µL of butanol:methanol (1:1, v/v) spiked with internal standards (IS). For leukocytes samples, 200 µL of butanol:methanol (1:1, v/v) was added directly to the pelleted leukocytes. For human plasma samples, ten µL of plasma were combined with 190 µL of butanol:methanol (1:1, v/v) spiked with internal standards (ISTD). The standards were purchased from Avanti Lipids and included acylcarnitine 16:0 D3, cholesterol ester 18:0 D6, dihydroceramide d18:0/08:0, ceramide d18:1/12:0, deoxyceramide m18:1/12:0, diacylglycerol 15:0/15:0, GM3 d18:1/18:0 D3, monohexosylceramide d18:1/12:0, dihexosylceramide d18:1/12:0, trihexosylceramide d18:1/18:0 D3, lysophosphatidylcholine 13:0, lysophosphatidylethanolamine 14:0, phosphatidylcholine 13:0/13:0, phosphatidylethanolamine 17:0/17:0, phosphatidylglycerol 17:0/17:0, phosphatidylinositol 12:0/13:0, phosphatidylserine 17:0/17:0, sphingomyelin d18:1/12:0, sphingosine d17:1, and triacylglycerol 12:0/12:0/12:0. In addition, monoacylglycerol 19, and sphinganine d17:0 were used for serum samples. Plasmalogen phosphatidylcholine 18:0/18:1 D9, plasmalogen phosphatidylethanolamine 18:0/18:1 D9, and cholesterol-D7 were for other endogenous lipid analysis. Internal standards for d82-DOPC tracing study were dihydroceramide d18:0/08:0, ceramide d18:1/12:0, deoxyceramide m18:1/12:0, diacylglycerol 15:0/15:0, monoacylglycerol 19:0, monohexosylceramide d18:1/12:0, dihexosylceramide d18:1/12:0, lysophosphatidylcholine 13:0, lysophosphatidylethanolamine 14:0, phosphatidylcholine 13:0/13:0, phosphatidylethanolamine 17:0/17:0, phosphatidylglycerol 17:0/17:0, phosphatidylinositol 12:0/13:0, phosphatidylserine 17:0/17:0, sphingomyelin d18:1/12:0, sphingosine d17:1, sphinganine d17:0, 13C stearic acid, and triacylglycerol 12:0/12:0/12:0. Phosphatidylglycerol 17:0/17:0 was used for normalization of the signal of BMP in the samples. The mixture was vortexed for 10 seconds, sonicated for 30

minS and then centrifuged at 4°C at14,000 xg for 10 mins. The supernatant fraction was collected for LC-MS/MS analysis. A pooled lipid extract was used as a quality control (QC) sample and injected every five study samples. Data were normalized to protein concentration in each sample. For lysosome fractions, fractions were diluted to the same initial volume (1 mL) and mixed with 1.1 mL of chloroform (spiked with the same internal standards described above) and 2.2 mL of methanol. The mixture was vortexed for 10 seconds and shaken for 5 mins. Then again 1.1 mL of chloroform were added, the mixture was vortexed for 10 seconds and mixed for 5 minutes. Water (1.1 mL) was added to each sample, the mixture was vortexed for 10 seconds, shaken for 5 mins and then centrifuged at 3,000 xg for 10 mins at 4°C. The same volume from the lower fraction was collected for all the samples and dried by Speedvac. Extracts were resuspended in 100 µL of butanol:methanol (1:1, v/v) and vortexed for 30 seconds before analysis by LC-MS/MS. A pooled lipid extract was used as a quality control (QC) sample and injected every five samples. Data were normalized to the protein concentration and original volume of each lysosome fraction.

LC-MS/MS analysis

The LC-MS/MS analysis was performed on an Agilent UHPLC 1290 Infinity II liquid chromatography system connected to an Agilent QqQ 6495C. An Agilent Zorbax RRHD Eclipse Plus C18 column (2.1 × 50 mm, 1.8 µm) was used for the RPLC separation. The mobile phases A (60% water and 40% acetonitrile with 10 mmol/L ammonium formate) and B (10% acetonitrile and 90% isopropanol with 10 mmol/L ammonium formate) were used for the chromatographic separation. The following gradient was applied: 0-2 min, 20-60% B; 2-12 min, 60-100% B; 12-14 min, 100% B; 14.01-15.8 min, 20% B. The oven temperature was maintained at 40°C. Flow rate was set at 0.4 mL/min and the sample injection volume was 2 or 5 µL, depending on the sample type. The positive ionization spray voltage and nozzle voltage were set at 3,000 V and 1,000 V, respectively. The drying gas and sheath gas temperatures were both maintained at 250 °C. The drying gas and sheath gas flow rates were 14 L/min and 11 L/min, respectively. The nebulizer nitrogen gas flow rate was set at 35 psi. The iFunnel high and low pressure RF were 150 V and 60 V, respectively.

SFC-MS-QTOF analysis

SFC-MS-QTOF analysis was performed for d82-POPC tracing study on an Agilent 6546 Q-TOF and an Agilent 1260 Infinity II SFC System. Lipid separation was performed on a VIRIDIS BEH (3 x 100 mm, 1.7 µm) column at 60 °C in the following conditions: autosampler temperature 10 °C, injection volume 1 µL, overfeed volume 0 µL, back pressure regulator (BPR) 100 Bar, BPR temperature 60 °C, modifier: methanol/water (99/1; *v/v*) with final concentration of ammonium acetate 30 mM, flow rate 1.8 mL/min, gradient: 0-1.5 min (1-16 % of modifier), 4-6 min (51 % of modifier), 6.10 min (1 % of modifier) with total run of 8 min. The same solvent mixture as for the modifier was used as a make-up solvent with a flow rate of 0.25 mL/min. The Q-TOF mass spectrometer was equipped with a Dual AJS-ESI ion source and operating under the following conditions: gas temperature 200 °C; gas flow 12 L/min; nebulizer 50 psi; sheath gas temperature 400 °C; sheath gas flow 12 L/min; capillary 5000 V. Untargeted analysis was performed in positive ion mode and MS1 full scan in the range of 150-1200 *m/z.*

LC-MS-QTOF analysis

The LC-MS-QTOF analysis was performed on an Agilent UHPLC 1290 Infinity liquid chromatography system connected to an Agilent 6546 Q-TOF. The separation and ion source parameters were the same as for LC-MS/MS analysis described above except for injection volume (5 µL). The analysis was performed using auto MS/MS in positive and negative mode with a mass range 100-1000 *m/z* (MS1) and 50-1000 *m/z* (MS2) at acquisition rates of 4 spectra/s and collision energy of 25 V.

Data Analysis of lipidomic data

The acquired MS data were analyzed using Agilent MassHunter software version 10.1, either using QqQ Quantitative Analysis or Q-TOF Quantitative analysis. The isotopic correction (in the case of SFC-MS-QTOF analysis) was done using LipidQuant 2.1 software. The signal to noise ratios (S/N) were calculated using the raw peak areas in both QC samples and processed blanks (PBLK). Lipids that had S/N < 10, CV > 20% in the QC samples and did not show a linear

behaviour (R^2 < 0.8) in dilution curves were excluded from further analysis. Internal standards were used to normalize the raw peak areas in the corresponding lipid class (one internal standard per class) and concentrations were further normalized to the protein concentration in the original sample.

Additional References

1. Katoh K, and Standley DM. MAFFT Multiple Sequence Alignment Software Version 7: Improvements in Performance and Usability. *Molecular Biology and Evolution.* 2013;30(4):772-80.
2. Waterhouse AM, Procter JB, Martin DMA, Clamp M, and Barton GJ. Jalview Version 2—a multiple sequence alignment editor and analysis workbench. *Bioinformatics.* 2009;25(9):1189-91.
3. Hannah VC, Ou J, Luong A, Goldstein JL, and Brown MS. Unsaturated fatty acids down-regulate srebp isoforms 1a and 1c by two mechanisms in HEK-293 cells. *J Biol Chem.* 2001;276(6):4365-72.
4. Schmidt A, Danyel M, Grundmann K, Brunet T, Klinkhammer H, Hsieh T-C, et al. Next-generation phenotyping integrated in a national framework for patients with ultrarare disorders improves genetic diagnostics and yields new molecular findings. *Nature Genetics.* 2024;56(8):1644-53.
5. Liu H, and Naismith JH. An efficient one-step site-directed deletion, insertion, single and multiple-site plasmid mutagenesis protocol. *BMC Biotechnol.* 2008;8:91.
6. Quek DQ, Nguyen LN, Fan H, and Silver DL. Structural Insights into the Transport Mechanism of the Human Sodium-dependent Lysophosphatidylcholine Transporter MFSD2A. *J Biol Chem.* 2016;291(18):9383-94.
7. Thelen M, Winter D, Braulke T, and Gieselmann V. SILAC-Based Comparative Proteomic Analysis of Lysosomes from Mammalian Cells Using LC-MS/MS. *Methods Mol Biol.* 2017;1594:1-18.
8. He M, Kuk ACY, Ding M, Chin CF, Galam DLA, Nah JM, et al. Spns1 is a lysophospholipid transporter mediating lysosomal phospholipid salvage. *Proceedings of the National Academy of Sciences.* 2022;119(40):e2210353119.

Supplementary Table 1

Summary of Clinical and laboratory findings

**Patient :**	**Skeletal muscle:**	**Heart:**	**Liver:**	**CNS:**
Family A, Patient A.II.1: Male, born 2008	Proximal, axial, pharyngeal, and distal weakness in upper extremities, abdomen, hands. Increased fatiguability.<br>Cannot walk on heels. Difficulty to swallow. Slight scoliosis (Cobb angle 17°).<br><br>p-CK 1200-2400 U/L (2-5 times upper normal reference level)<br>p-Myoglobin 646-803 μg/L (9 -11 times upper normal reference level). EMG normal.<br><br>Muscle biopsy: normal morphology. No increase of lysosomes or peroxisomes.	Echocardiography: Increased trabeculation left ventricle. Discrete left ventricle hypertrophy. Normal systolic and diastolic function. ECG normal.	Neonatal transient non-conjugated hyperbilirubimemia.<br>s-ALAT 480-660 U/L (16-21 times upper normal reference level).<br>s-ASAT 412-509 U/L<br>Liver scan: slight to medium fibrosis. Normal values of p-ALP, p-albumin, p-bilirubin, PK, APTT, p-GT.<br><br>Liver biopsy: normal morphology. No deposits of glycogen or other material.<br>Electron microscopy: Increased and dilated ER. Disrupted structure of mitochondrial cristae. Deranged mitochondria with crystalline deposits.	Attention deficit disorder (ADD).<br>Cognitive skills at lower average to borderline range.
Family A, Patient A.II.2: Male, born 2016	Increased fatiguability. Normal gross motor function.<br><br>p-CK 420-1500 U/L (1-3 times upper normal reference level)<br><br>Muscle biopsy not performed.	Echocardiography: discrete signs increased trabeculation left ventricle. Normal systolic and diastolic function. ECG normal.	s-ALAT: 240-540 U/L (8-17 times upper normal reference level).<br>Normal values of p-ALP, p-albumin, p-bilirubin, PK, APTT, p-GT.<br><br>Ultrasound liver normal.<br>Liver biopsy not performed.	Late language development.

Family B,<br>Patient 3<br>Male, Born<br>Dec 2016	p-CK: 700-1000U/L<br>LDH:1200-1500U/L<br>Myoglobin: 481<br>Muscle biopsy not preformed	Prenatal suspicion of anomalous pulmonary vein opening<br>Cardiac ultrasound after birth showed dilated superior Vena cava, cardiomegaly with large coronary arteries, a large PDA, a small aortic isthmus stenosis but was normal in the subsequent visit	Elevation of transaminase first noted at age of 2.5 years old<br>p-ALAT: 220-640U/L<br>p-ASAT:150-370U/L<br><br>Ultrasound liver showed hepatopathy	At age of 3, lower vocabulary (approx. 10 Words)<br>Slightly hypotonic, increased patellar stretch reflex on both sides<br>Inconspicuous EEG and cranial MRI
