# Supplementary material for: SPNS1 variants cause multiorgan disease and implicate lysophospholipid transport as critical for mTOR-regulated lipid homeostasis
Source: J Clin Invest. 2025 Jul 3;135(17):e193099. doi: 10.1172/JCI193099 (PMC12404768; doi:10.1172/JCI193099)

**Full unedited blot for Figure 1A**

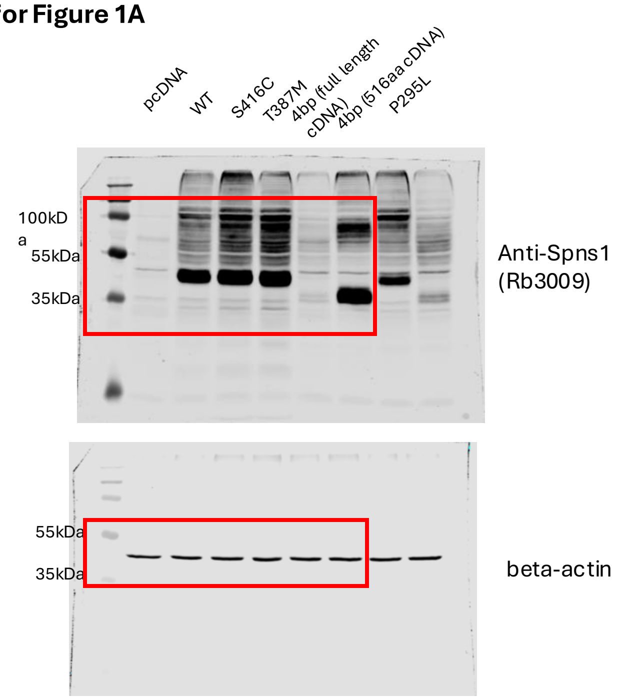


**Full unedited blot for Figure 1B, and replicates blot**

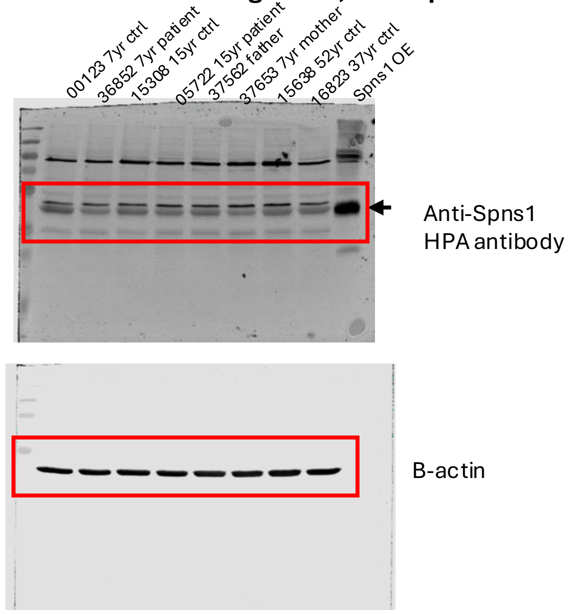


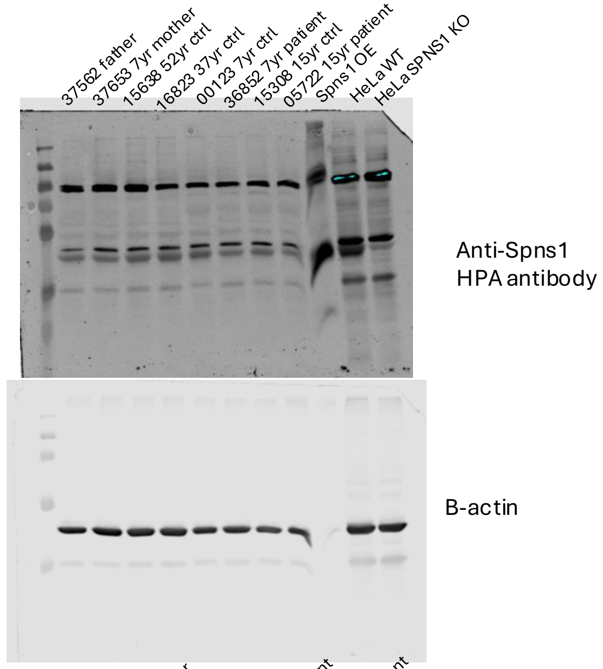


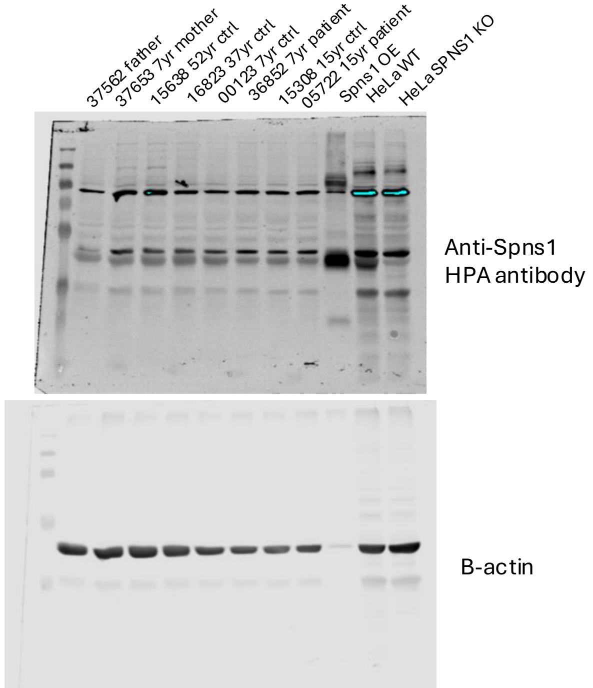


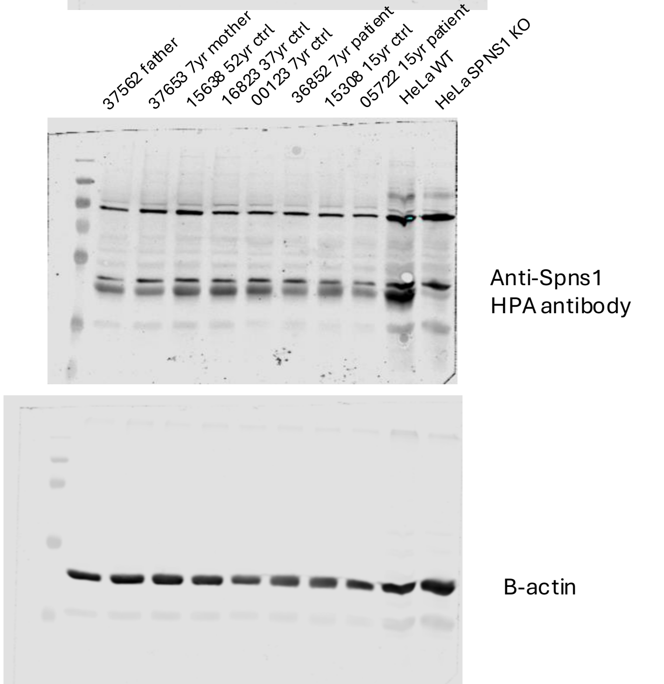

**Full thin layer chromatograph (TLC) for Figure 2F, and replicates**

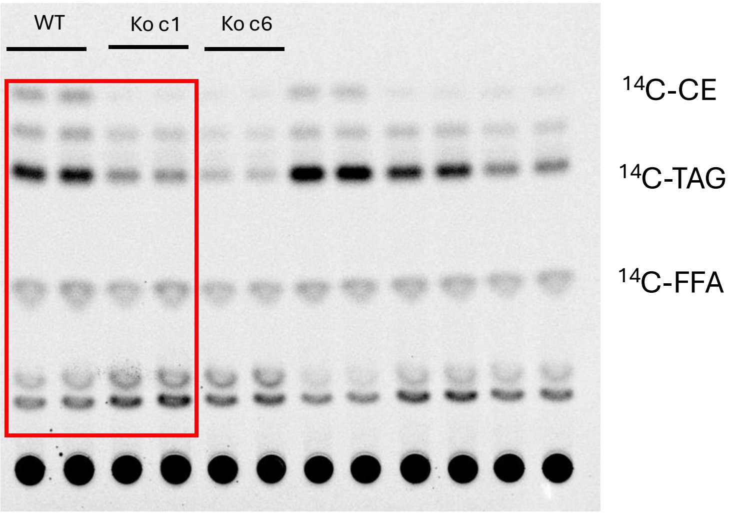


**Full thin layer chromatograph (TLC) for Figure 2H, and replicates**

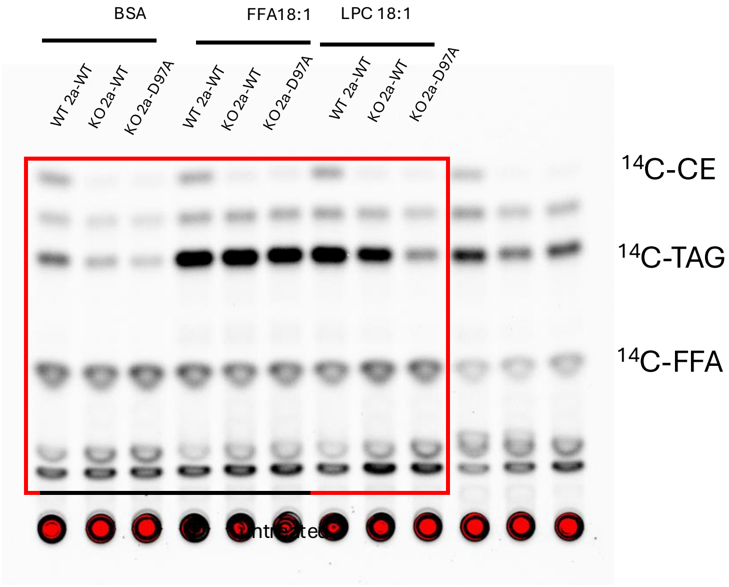


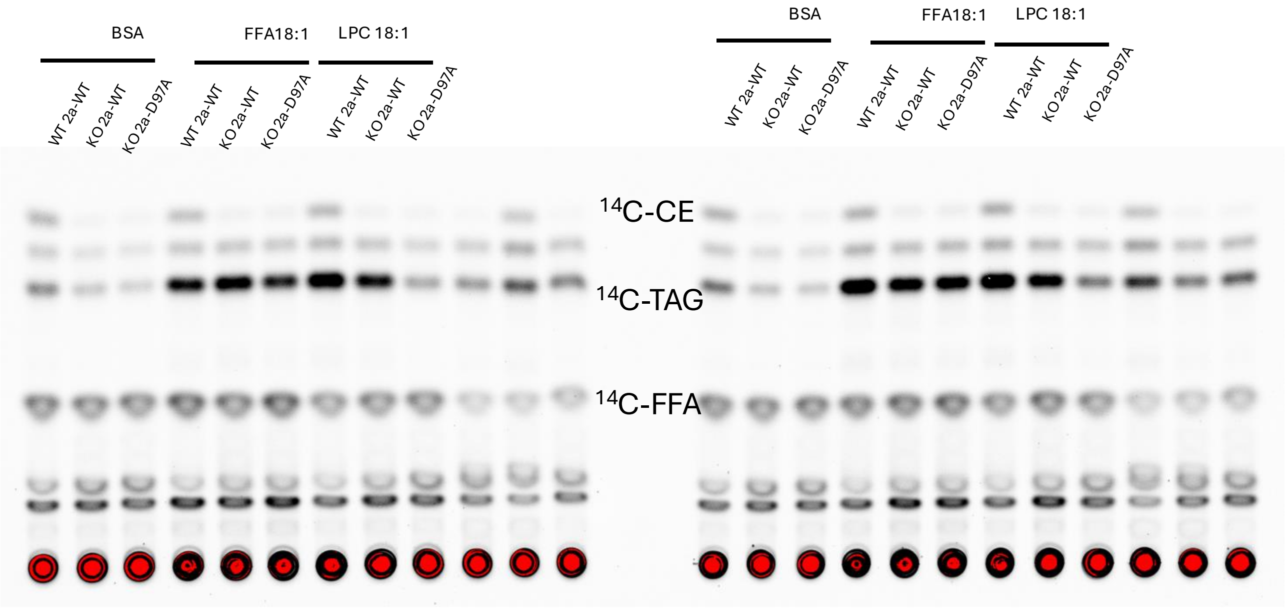

**Full unedited TLC for figure 4A**

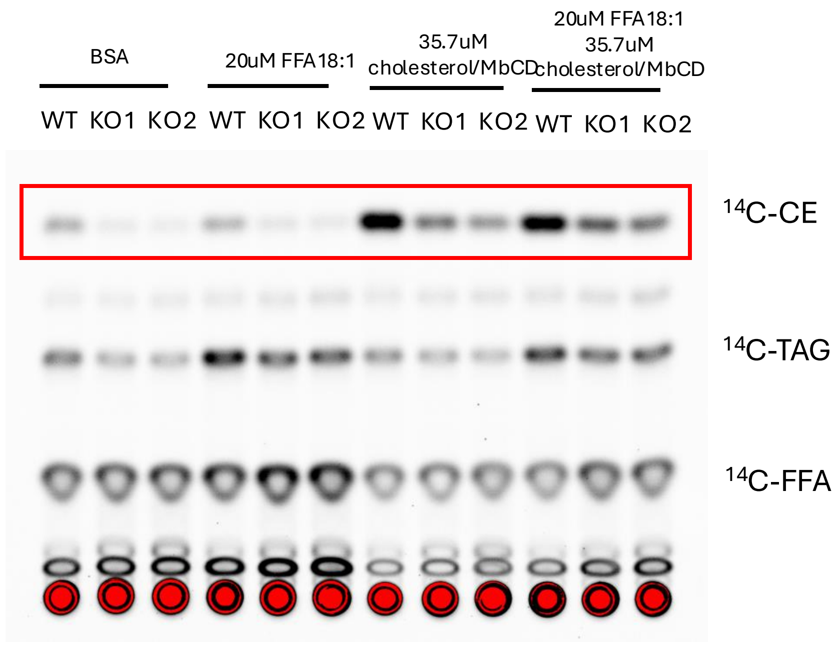


**Full unedited blot for Figure 4K**

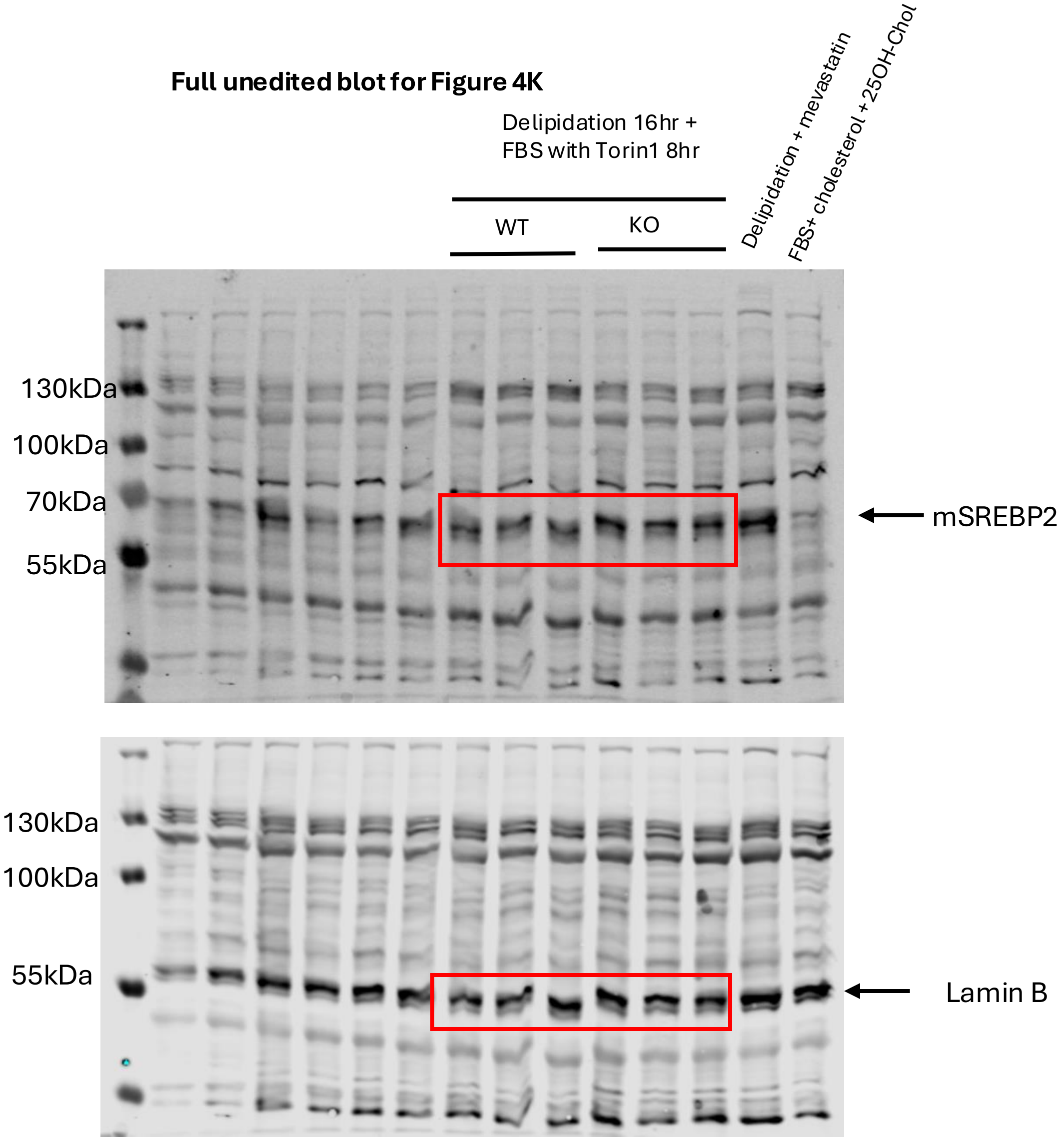

**Full unedited blot for supplemental Figure 1E, with replicates**

I: input
B: Bound

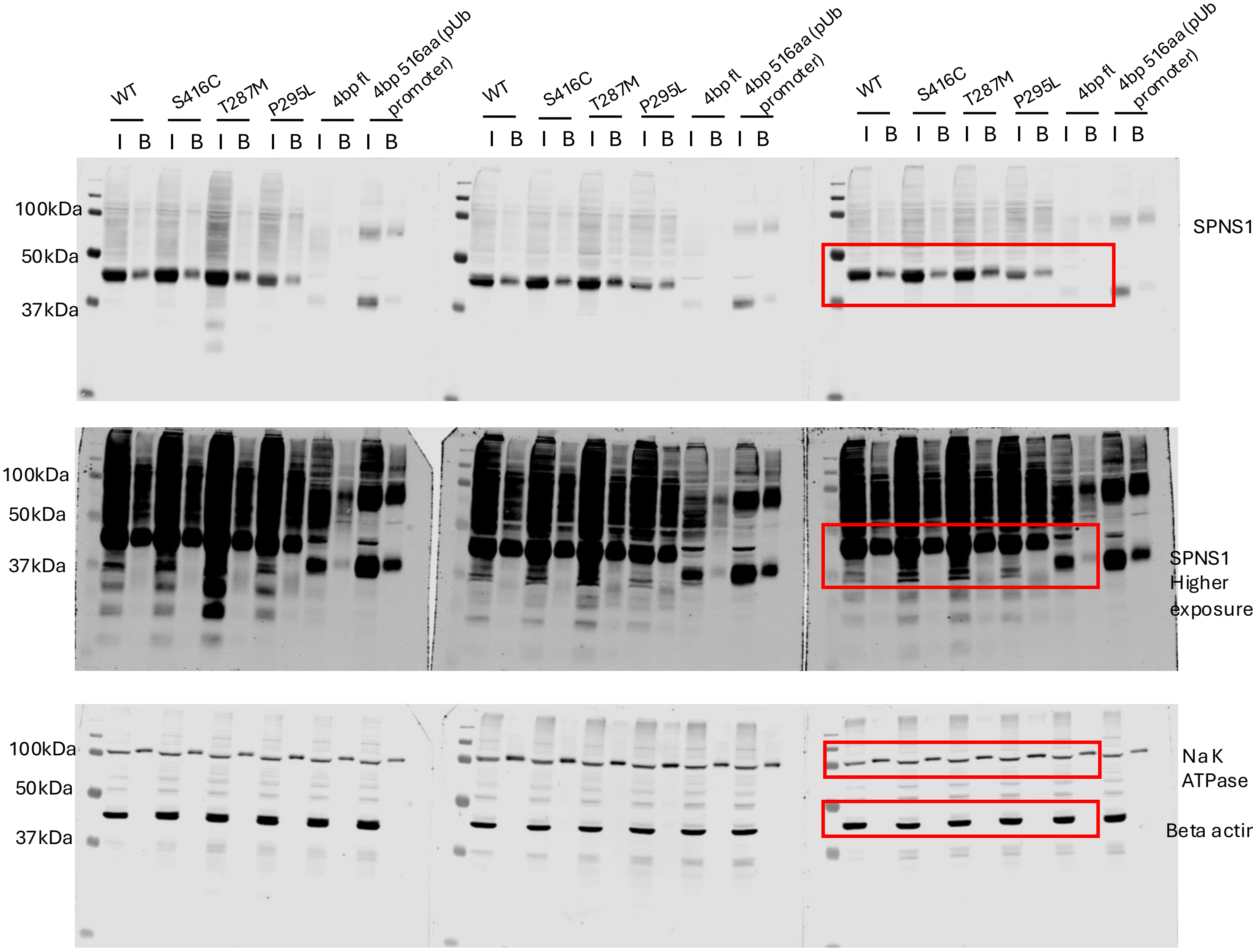

## Full unedited blot for supplemental Figure 1F

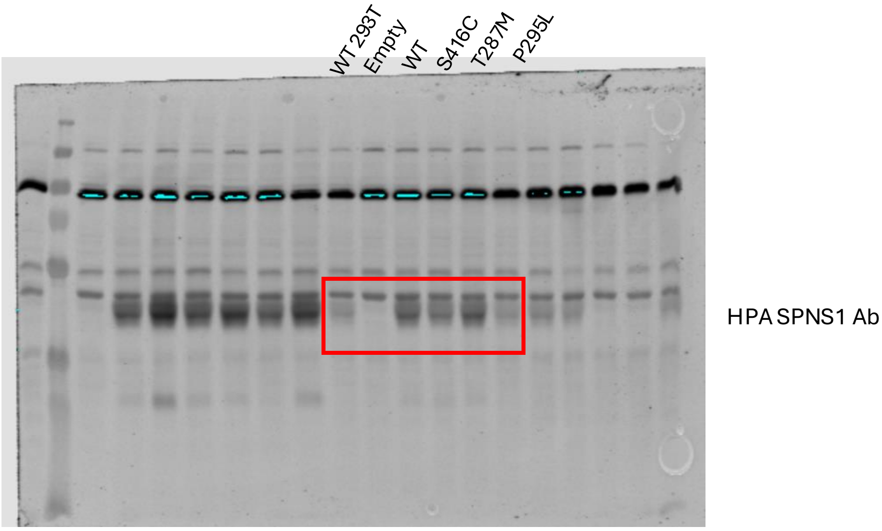


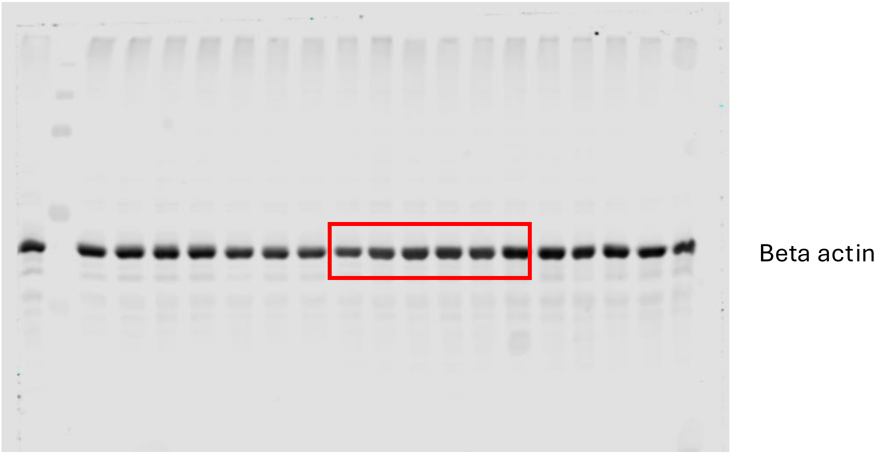


## Full unedited blot for supplemental Figure 1J

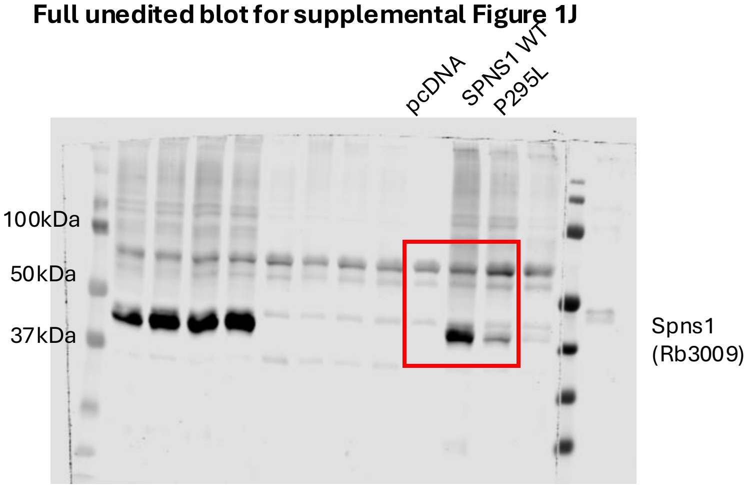


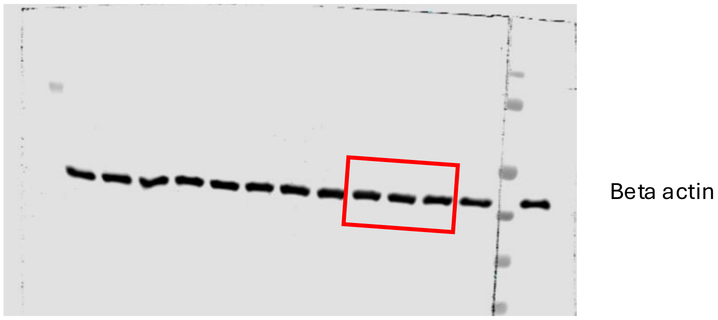

**Full unedited blot for supplemental Figure 2F**

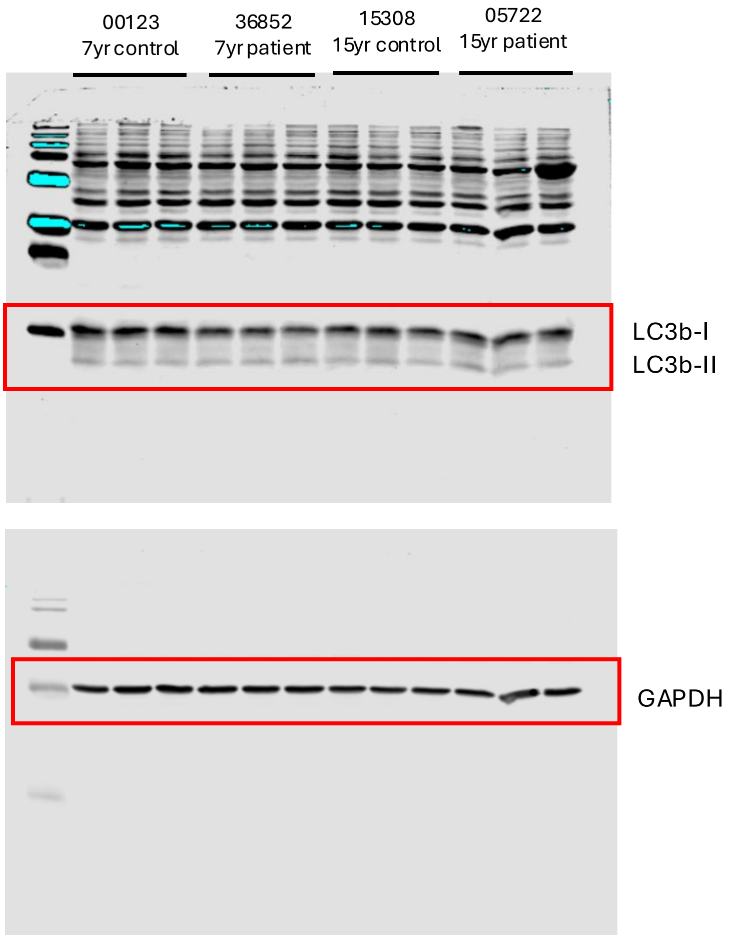

**Full unedited TLC for Supplemental figure 3C and replicates**

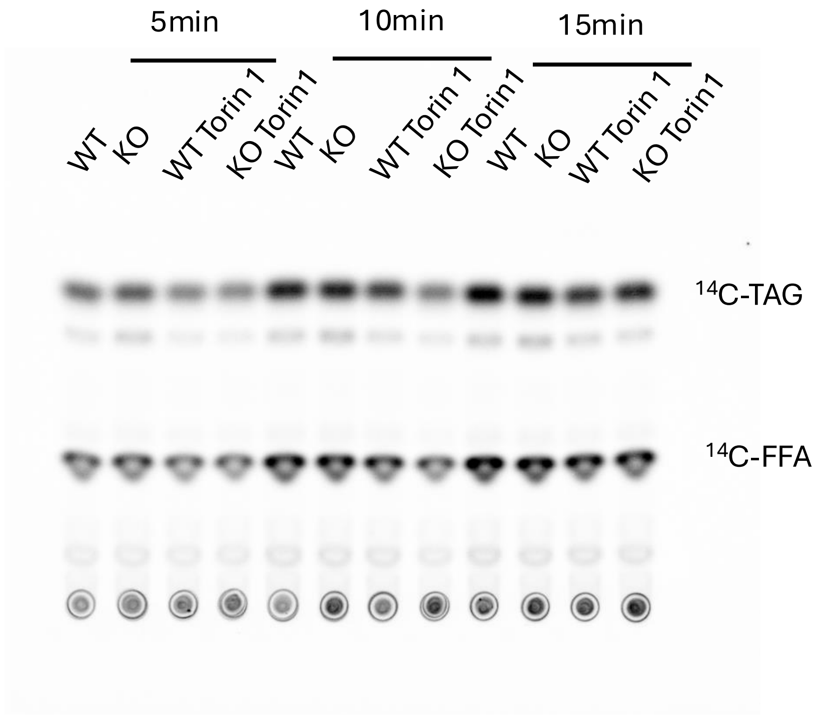


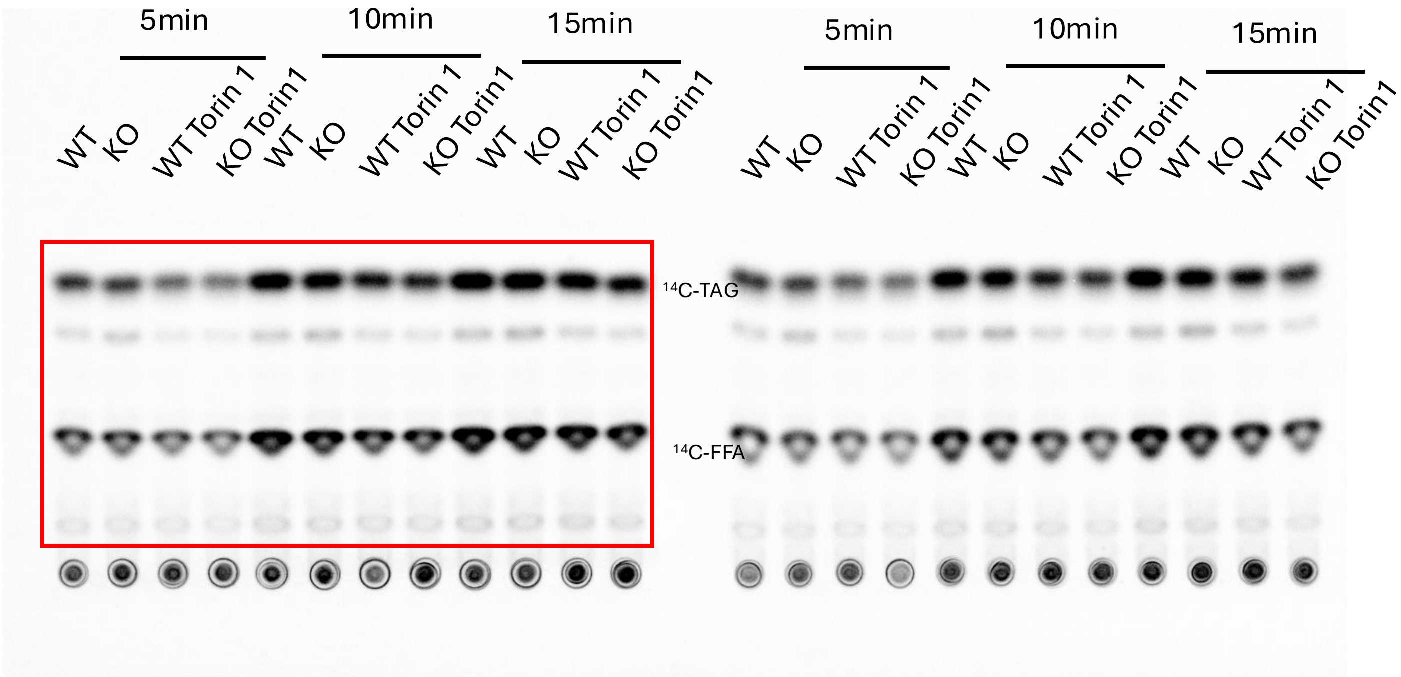

**Full unedited TLC for Supplemental figure 5G**

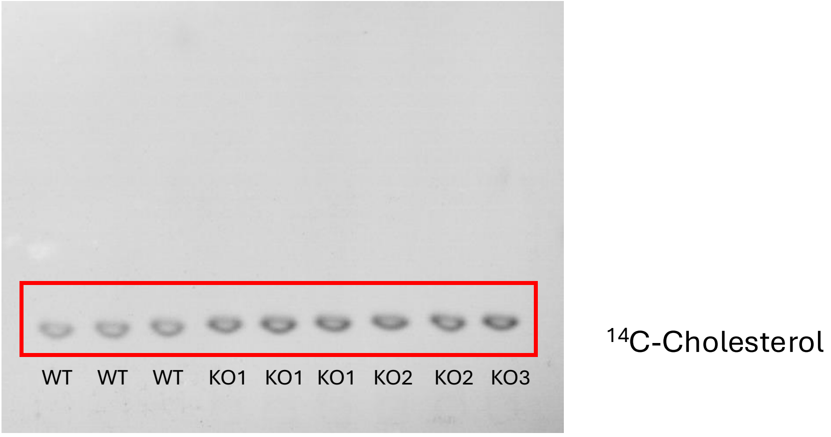


**Full unedited TLC for Supplemental figure 6A**

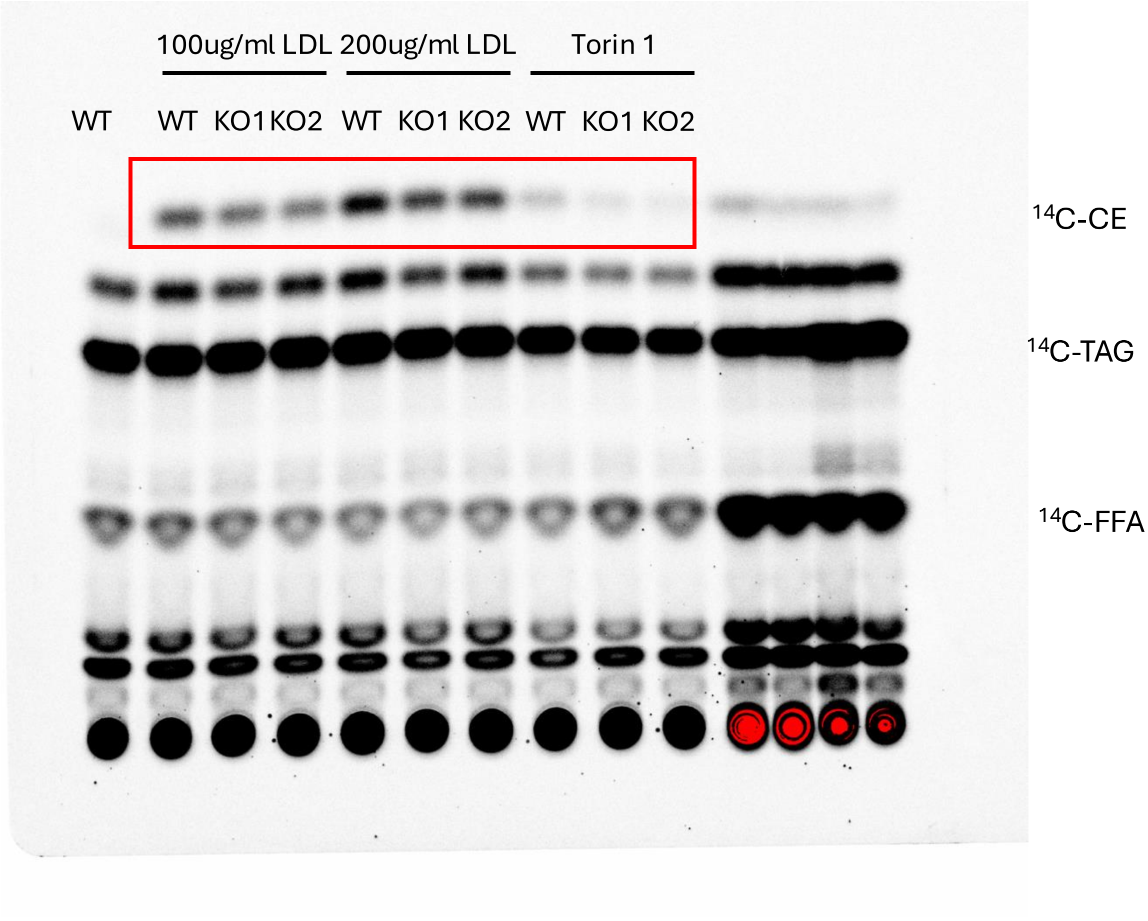

**Full unedited blot for supplemental Figure 7B**

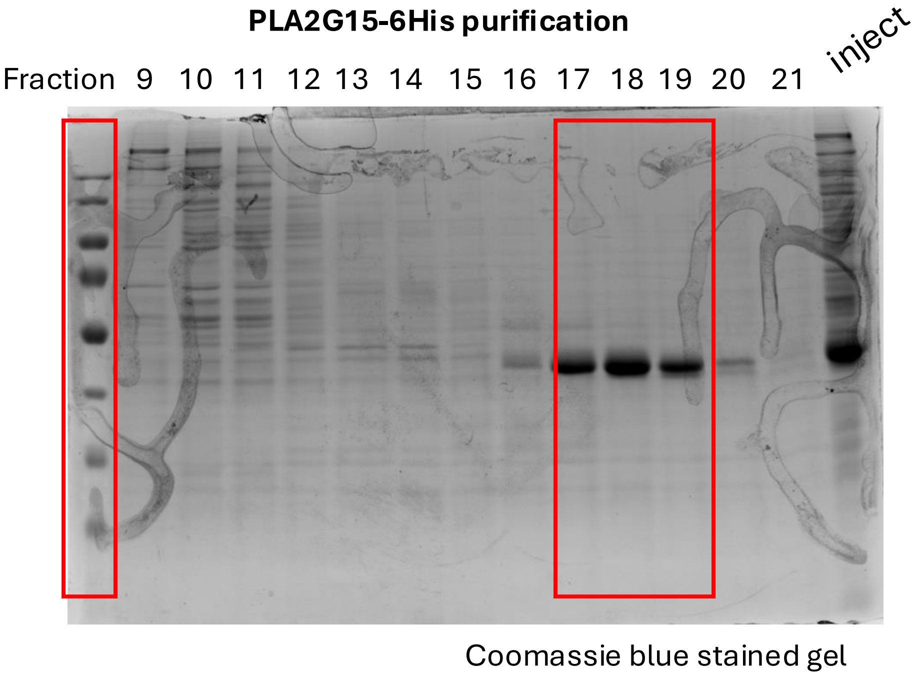


**Full unedited blot for supplemental Figure 7C**

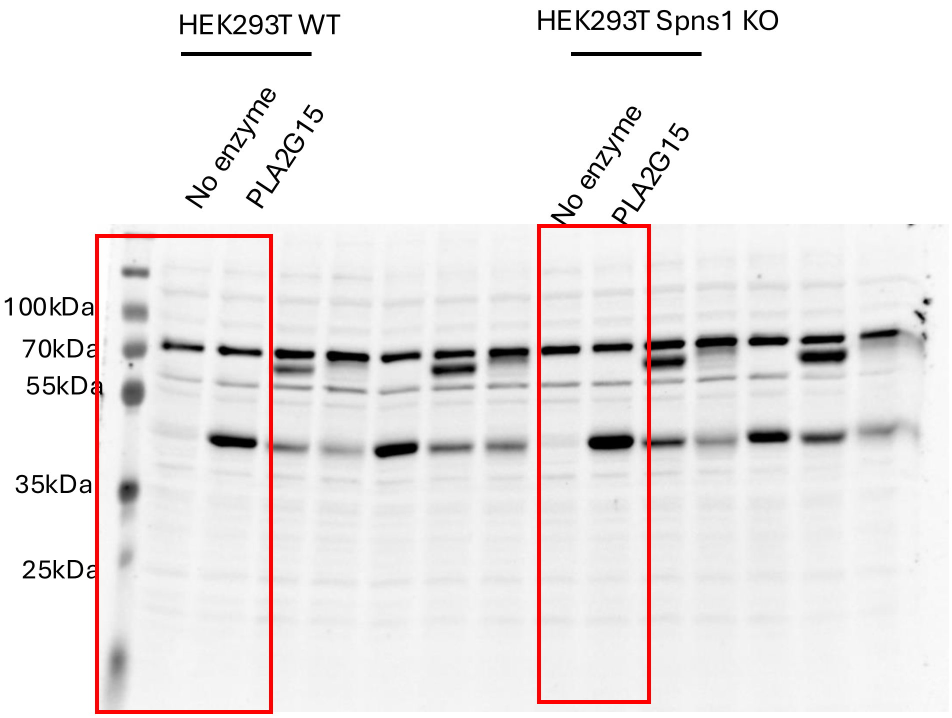

Supplement: Unedited blot and gel images [file jci-135-193099-s043.pdf]
